# Copper Sulfate Elicitation Effect on Biomass Production, Phenolic Compounds Accumulation, and Antioxidant Activity of *Morus nigra* L. Stem Node Culture

**DOI:** 10.3390/plants14050766

**Published:** 2025-03-02

**Authors:** Jan Senekovič, Špela Jelen, Andreja Urbanek Krajnc

**Affiliations:** Faculty of Agriculture and Life Sciences, University of Maribor, Pivola 10, 2311 Hoče, Slovenia; jan.senekovic1@um.si (J.S.); spela.jelen3@um.si (Š.J.)

**Keywords:** antioxidative potential, black mulberry, copper stress, elicitation of phenolics, flavonoids, in vitro culture, nodal culture, phenolic acids

## Abstract

Phenolic compounds are strong antioxidant and antibacterial agents with great pharmacological, medicinal, nutritional, and industrial value. The potential of *Morus nigra* in stem node culture was investigated for the production of phenolic compounds and their elicitation with CuSO_4_. Individual phenolic compounds in the samples were identified and quantified by using HPLC-PDA and HPLC-MS methods, while the content of total phenolic compounds, the content of total flavonoids, and the antioxidant activity of methanolic extracts were evaluated spectrophotometrically. The highest fresh and dry weights were obtained in plantlets treated with 0.5 mM CuSO_4_ for 42 days. The highest total phenolic content, total flavonoid content, and antioxidant activity of the extracts were determined in stem node cultures treated with 3 mM CuSO_4_ for 42 days. Under the latter conditions, the predominant representatives of the caffeoylquinic acids, *p*-coumaric acid derivatives, kaempferol derivatives, and quercetin derivatives also achieved the highest content. The most abundant phenolic compound in all samples was the chlorogenic acid. The nodal culture of *M. nigra* elicited with CuSO_4_ could potentially be used for the industrial production of phenolic compounds, especially caffeoylquinic acids. Moreover, considering the biochemical response to CuSO_4_ treatment and the ability to tolerate and accumulate copper, the potential application of *M. nigra* in phytoremediation is also highlighted.

## 1. Introduction

Phenolic compounds are a vast class of secondary metabolites performing several roles for the defense and survival of plants [1]. All phenolic compounds contain at least one aromatic ring with one hydroxyl group in their structure. There are more than 8000 individual plant phenolic compounds, with great structural variability [2]. Two fundamental metabolic pathways are involved in the biosynthesis of phenolic compounds in plants—the shikimate pathway and the malonate pathway—which is less significant in higher plants [3]. The function of phenolic compounds in plants is primarily to defend against herbivores, pathogens, and abiotic stress such as photothermal stress, unfavorable temperature and pH, saline stress, exposure to higher levels of carbon dioxide, exposure to higher levels of ozone, and heavy metal stress [4]. Phenolic compounds exhibit diverse bioactivities, including antioxidant activities, antimicrobial activities, anti-inflammatory properties, and antiproliferative activities against cancerous and tumor cells [5]. A long-term consumption of diets high in plant polyphenols protects against cancer, cardiovascular diseases, diabetes, osteoporosis, and neurological diseases [6]. Moreover, phenolic compounds also possess great potential to be used as preservatives, replacing the synthetic ones in different food and cosmetic products [7]. They are highly valuable compounds due to their potential utilization as natural, bioactive, and antioxidant molecules for the food, cosmetic, chemical, and pharmaceutical industries [8]. Flavonoids and phenolic acids are the most interesting groups of phenolic compounds in plants [9]. Due to the relatively low contents and long growth cycle of cultivated resource plants, the production of phenolic acids and flavonoids cannot meet the fast-growing market needs [10]. Various biotechnological applications have been utilized to improve phenolic compound production [11]. The in vitro culture technique appears as an environmentally friendly alternative technique for the production of secondary metabolites when natural supply is limited, and traditional methods are unfeasible [12]. Obtaining the desired metabolites using the in vitro culture technique has many advantages. These are as follows: tasks such as watering, spraying, and weeding can be skipped; production is continuous and independent of the season; it allows an increase in efficiency by using biotransformation methods; it can be used in large-scale production; it enables the production of a large number of clones with the desired characteristics; optimization and usage of elicitors is possible and is independent of climate and soil changes [13].

Elicitation is a technique that involves the exogenous addition of elicitors (abiotic or biotic) in the growth medium, which consequently triggers a stress response with concomitant enhancement in secondary metabolite production [14]. Heavy metals, such as Ag, Cd, Co, Cu, Fe, and Ni can act as abiotic elicitors for the production of secondary metabolites [15]. Exposure to heavy metals increases the production of phenolic compounds in plants. They are important antioxidants and metal chelators and have been considered electron-donating agents. The protective role of plant phenolic compounds could explain the modulation of their levels, depending on the stress conditions in the environment [16]. The biosynthesis of phenolics under stressful environments is regulated by the altered activities of various key enzymes of the phenolic biosynthetic pathways like phenylalanine ammonia lyase (PAL) and chalcone synthase (CHS). The enhanced performance of enzymes is also accompanied by the up-regulation of the transcript levels of genes encoding key biosynthetic enzymes [17]. Copper is an essential metal for normal plant growth and development, although it is potentially toxic [18]. It participates in a number of metabolic processes such as mitochondrial respiration, hormone signaling, photosynthetic electron transport, cell wall metabolism, and superoxide scavenging. It also functions as a structural component in a variety of enzymes, including cytochrome c oxidase, laccase, amino oxidase, plastocyanin, polyphenol oxidases (PPO), and Cu/Zn superoxide dismutase [19]. When Cu is presented in high amounts (in some plant species, amounts already higher than 200 μM), it causes cytotoxic injuries to plants. This results in a hindrance of plant growth and causes the chlorosis of leaf margins. Copper toxicity adversely influences the growth and oxidative mechanisms of plants [20]. Copper treatment leads to the activation of the phenylpropanoid pathway [21]. Enzymes and genes related to the phenolic biosynthesis pathway are involved in the response to copper stress [22]. It was proven that copper stress significantly increases the activity of PAL [23]. Consequently, the phenolic level increases significantly [16,24,25]. Therefore, copper sulfate (CuSO_4_) is a promising elicitor for the accumulation of phenolic compounds in in vitro cultures [26].

Mulberry is the most medicinally important plant of the genus *Morus* [27]. Many studies have shown that mulberry leaves exhibit a wide range of pharmacological activities [28]. Mulberries have been gaining increasing attention due to their health-promoting effects, including anti-diabetic, anti-obesity, anti-hyperglycemic, anti-hypercholesterolemic, anti-inflammatory, and gut microbiota modulatory activities [29]. Recent reports indicate that mulberry leaves are a rich source of polyphenolic substances, including phenolic acids and flavonoids such as *p*-coumaric acid derivatives, caffeoylquinic acids, kaempferol derivatives, and quercetin derivatives [30,31,32,33,34,35,36,37]. *Morus alba* L. (white mulberry), *M. nigra* L. (black mulberry), and *M. rubra* L. (red mulberry) species are the most widespread species from the genus *Morus* [38]. Compared to white mulberry leaves, black mulberry leaves contain a higher amount of phenolic compounds [39,40,41,42,43,44]. In vitro cultivation is a proven and successful method of *M. nigra* propagation. Also, there are already successful studies that investigated the in vitro cultures of black mulberry using various sterilization methods and growth regulators combinations [45,46]. Based on the aforementioned studies which state that *M. nigra* is a rich source of phenolics, exhibits positive health effects, and thrives in plant in vitro cultures, it is assumed that stem node culture exhibits potential for the production of phenolic compounds.

The aim of this study is to evaluate the profile of phenolic acids and flavonoids along with the antioxidant activity of *M. nigra* stem node culture. In addition, this study will also evaluate the elicitation potential of *M. nigra* stem node culture with CuSO_4_ (0.5 mM; 1 mM; 3 mM) and its influence on the production of fresh and dry weight, the content of total and individual phenolic compounds, and the antioxidant activity of the extracts. To the best of our knowledge, this is the first study which examines the phenolic compound compositions in black mulberry stem node culture and the effect of CuSO_4_ elicitation on it.

## 2. Results

### 2.1. Influence of CuSO_4_ Treatment on Plantlets’ Development

**Figure 1:**
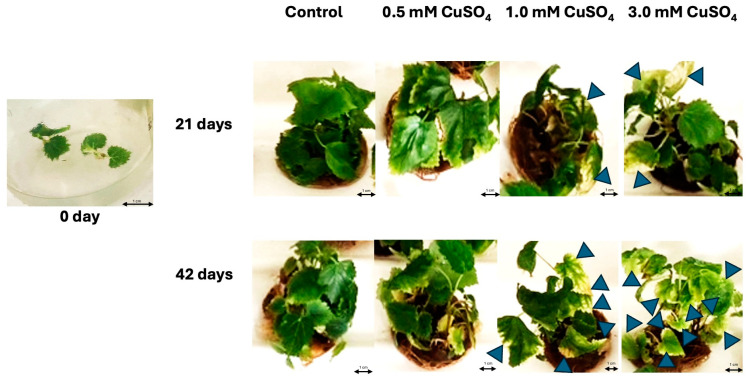
Morphologically visible changes in the stem node culture of *M. nigra* treated with different CuSO_4_ treatments. Arrowheads indicate where leaf marginal chloroses formed.

Morphologically noticeable changes in stem node cultures treated with different concentrations of CuSO_4_ and grown for different periods of time are shown in Figure 1. At 0 day of the experiment, before the stem node cultures were treated with CuSO_4_, all plantlets included in the experiment were approximately the same size. Their leaves were bright green, without visible necrosis, chlorosis, or deformations. Visible morphological changes between tested treatments were already observed at the first observation of the plantlets 21 days after the beginning of the experiment. Plantlets to which CuSO_4_ was not added (control group) had bright green leaves without visible necrosis, chlorosis, or deformations. Plantlets treated with the 0.5 mM CuSO_4_ solution were slightly larger than plantlets from the control group. They also had bright green leaves without visible morphological changes. Plantlets treated with the 1 mM CuSO_4_ solution were similar in size to plantlets from the control group. On a smaller number of their leaves, small circular marginal chlorosis appeared and spread toward the center of the leaf. The plantlets that were exposed to the treatment with the 3 mM CuSO_4_ solution were smaller in size than the plantlets from the control group. Numerous circular marginal chloroses were observed on the leaves, spreading toward the center of the leaf. Even greater morphologically noticeable changes between stem node cultures grown and treated with different concentrations of CuSO_4_ were observed 42 days after the start of the experiment. Even after 42 days of the experiment, plantlets from the control group had bright green leaves without the presence of visible chlorosis, necrosis, and deformations. Plantlets treated with the 0.5 mM CuSO_4_ solution were slightly larger compared to plantlets from the control group and showed no visible changes in the leaves. Plantlets grown in MS medium supplemented with the 1 mM CuSO_4_ solution were visibly smaller compared to plantlets from the control group. Around half of their leaves contained small to medium-sized circular marginal chlorosis. Among all the treatments tested, after 42 days, nodal cultures supplemented with the 3 mM CuSO_4_ solution were visibly the smallest. On their leaves, medium-sized to large marginal chloroses were observed. Some marginal chloroses spread over the entire leaf.

### 2.2. Influence of CuSO_4_ Treatment on Fresh and Dry Weight

**Figure 2:**
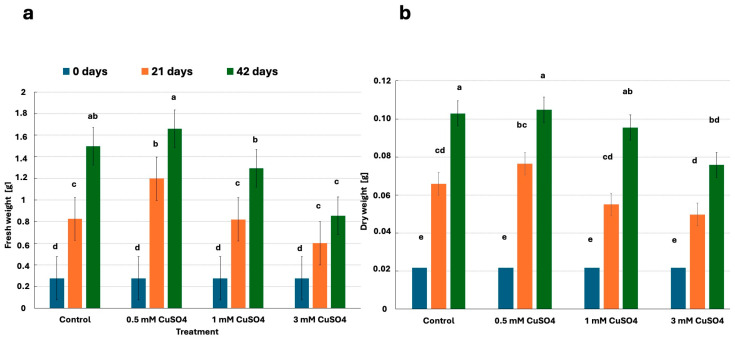
The effect of different CuSO_4_ treatments on the average ± SD (**a**) fresh weight and (**b**) dry weight of *M. nigra* plantlets in vitro after 21 and 42 days of the experiment. Significant differences are indicated by different letters (post hoc Duncan test, *p* < 0.05, n = 24).

There were statistically significant differences (*p* < 0.05, post hoc Duncan test) in average fresh and dry weights among plantlets cultivated under differently tested CuSO_4_ treatments. The most successful growth was observed in plantlets treated with the 0.5 mM CuSO_4_ solution throughout the entire 42 days of the experiment (Figure 1 and Figure 2). The mass (fresh weight 0.2754 g; dry weight 0.0217 g) of plantlets treated with the 0.5 mM CuSO_4_ solution increased by four times (fresh weight 1.1979 g; dry weight 0.0758 g) after 21 days and by six times (fresh weight 1.6601 g; dry weight 0.1048 g) after 42 days compared to 0 days of the experiment. A similar trend of changes in fresh and dry weight was also observed in the control group. The weight of the plantlets grown in the medium without the addition of CuSO_4_ increased approx. three times during (fresh weight 0.8265 g; dry weight 0.0658 g) the 21 days of the experiment and approx. five times (fresh weight 1.4967 g; dry weight 0.1029 g) during the 42 days of the experiment. A very similar trend of increasing fresh and dry weight as in the control was observed in cultures treated with 1mM CuSO_4_. In the first 21 days, their mass increased by approx. 3 times (fresh weight 0.8200 g; dry weight 0.0550 g), while in 42 days, it increased by approx. 4.5 times (fresh weight 1.2952 g; dry weight 0.0955 g). Plantlets treated with 3 mM CuSO_4_ gained fresh and dry weight the slowest throughout the experiment. During the 21 days of the experiment, their weight increased by approx. two times (fresh weight 0.6011 g; dry weight 0.0498 g), and during the 42 days of the experiment, by about three times (fresh weight 0.8555 g; dry weight 0.0758 g).

### 2.3. Influence of CuSO_4_ Treatment on Total Phenolic Content and Total Flavonoid Content

The highest total phenolic content (TPC) and the highest total flavonoid content (TFC) were observed in plantlets treated with 3 mM CuSO_4_ (Table 1). The concentration range of TPC in *M. nigra* stem node culture treated with different concentrations of CuSO_4_ was between 6.943 mg and 14.249 mg of gallic acid equivalent (GA)/g DW. TPC differed statistically significantly among most experimental systems (Table 1 and Table S1). In plantlets treated with 0.5 mM, 1.0 mM, and 3.0 mM CuSO_4_, the TPC values increased throughout the 42 days of the experiment. A different trend in TPC changes was observed in plantlets grown under control conditions. After 21 days of the experiment, the TPC value in these plants decreased compared to day 0 of the experiment, while after 42 days, compared to day 0, the TPC value increased. After 21 days of the experiment, as well as after 42 days of the experiment, higher TPC was achieved by plantlets treated with higher concentrations of CuSO_4_.

TFC differed significantly between all the experimental systems (Table 1 and Table S1). Its concentration range was between 0.697 mg and 1.216 mg of rutin equivalent (RUT)/g DW. The content of total flavonoids increased in plantlets grown under control conditions and in plantlets treated with 1 mM and 3 mM CuSO_4_ throughout the 42 days of the experiment. A different trend in the change in TFC values was detected in plantlets treated with 0.5 mM CuSO_4_. In aforementioned plantlets, the TFC values decreased after 21 days of the experiment compared to 0 days of the experiment and increased after 42 days compared to day 0. Plantlets treated with higher concentrations of CuSO_4_ achieved higher TFC after 21 and after 42 days of experiment.

### 2.4. Qualitative Analysis of Phenolic Compounds from M. nigra Stem Node Culture

**Figure 3:**
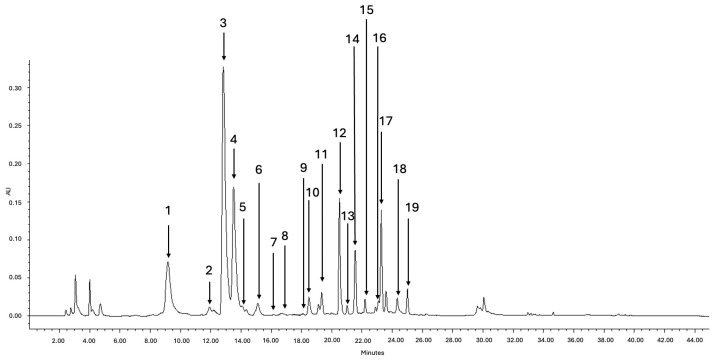
HPLC-PDA chromatogram of the acidified methanolic extract of *M. nigra* stem node culture treated with the most efficient 3 mM CuSO_4_ treatment for phenolic compound syntheses recorded at max. plot (λ = 280-350 nm) under optimal chromatographic conditions: (1) *p*-coumaric hexoside 1; (2) *p*-coumaric acid hexoside 2; (3) chlorogenic acid; (4) 4-caffeoylquinic acid; (5) *cis*-5-caffeoylquinic acid; (6) *trans*-5-*p*-coumaroylquinic acid; (7) *cis*-5-*p*-coumaroylquinic acid; (8) 3-*p*-coumaroylquinic acid; (9) quercetin dirhamnosyl-hexoside; (10) kaempferol dirhamnosyl-hexoside; (11) quercetin rhamnosyl-hexoside; (12) rutin; (13) quercetin-3-glucoside; (14) quercetin acetyl-rhamnosyl-hexoside; (15) kaempferol rhamnosyl-hexoside; (16) kaempferol acetyl-rhamnosyl-hexoside; (17) quercetin malonyl-hexoside; (18) quercetin acetyl-hexoside; (19) kaempferol acetyl-hexoside.

Qualitative and quantitative differences in the content of phenolic compounds in the stem node culture of *M. nigra* treated with different CuSO_4_ treatments were studied. A total of 19 phenolic compounds were identified (Figure 3). Five different *p*-coumaric acid derivatives, three caffeoylquinic acids, four kaempferol derivatives, and seven quercetin derivatives were identified by HPLC-PDA and HPLC-MS and quantified in all obtained extracts (Figure 3). Among *p*-coumaric acid derivatives, *p*-coumaric acid hexoside 1, *p*-coumaric acid hexoside 2, *trans*-5-*p*-coumaroylquinic acid, *cis*-5-*p*-coumaroylquinic acid, and 3-*p*-coumaroylquinic acid were identified in *M. nigra* nodal culture. Among the caffeoylquinic acids, chlorogenic acid, 4-caffeoylquinic acid, and *cis*-5-caffeoylquinic acid were determined.

Among the flavonoids, kaempferol derivatives and quercetin derivatives were identified. Kaempferol derivatives identified in the stem node culture of *M. nigra* were kaempferol dirhamnosyl-hexoside, kaempferol rhamnosyl-hexoside, kaempferol acetyl-rhamnosyl-hexoside, and kaempferol acetyl-hexoside. Among the quercetin derivatives, quercetin dirhamnosyl-hexoside, quercetin rhamnosyl-hexoside, rutin, quercetin-3-glucoside, quercetin acetyl-rhamnosyl-hexoside, quercetin malonyl-hexoside, and quercetin acetyl-hexoside were identified. More detailed data on the identified phenolic acids and flavonoids are presented in Table 2.

### 2.5. Influence of CuSO_4_ Treatment on Phenolic Acid and Flavonoid Content

**Figure 4:**
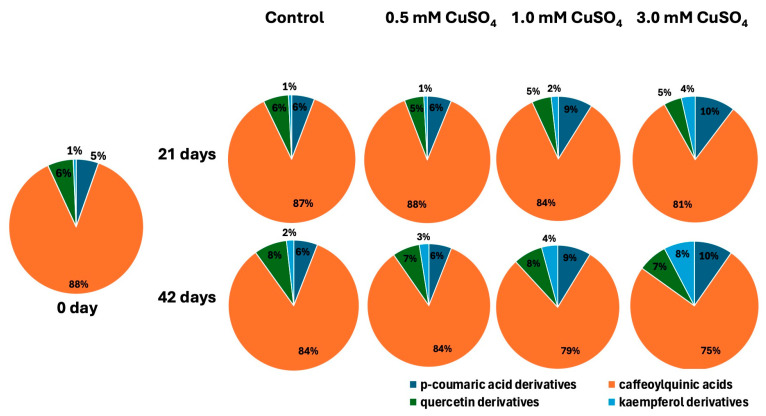
The proportion of the content of phenolic acid derivatives and flavonoid derivatives in *M. nigra* stem node culture treated with different concentrations of CuSO_4_ for different periods of time presented as pie charts.

At the beginning of the experiment (day 0), the majority of identified phenolic compounds in *M. nigra* stem node culture were caffeoylquinic acids (88%), followed by quercetin derivatives (6%), *p*-coumaric acid derivatives (5%), and kaempferol derivatives (1%) (Figure 4). After 21 days of the experiment, the proportion of phenolic compounds in the control group and in the group treated with 0.5 mM CuSO_4_ was similar to that at day 0 of the experiment. By increasing the concentration of treatment with CuSO_4_ (1 mM CuSO_4_ and 3 mM CuSO_4_), after 21 days of the experiment, it was observed that the proportion of *p*-coumaric acid derivatives and the proportion of kaempferol derivatives slightly increased, while the proportion of caffeoylquinic acids slightly decreased. The proportion of quercetin derivatives did not change between CuSO_4_ treatments after 21 days of the experiment.

After 42 days of the experiment, a slightly higher proportion of *p*-coumaric acid derivatives, kaempferol derivatives, and quercetin derivatives was observed in the control group compared to day 0 of the experiment. Despite this, caffeoylquinic acids represented the vast majority of the share of phenolic compounds determined in *M. nigra* nodal culture. By increasing the concentration of added CuSO_4_ after 42 days of the experiment, an increase in the proportion of *p*-coumaric acid derivatives and kaempferol derivatives, and a decrease in the proportion of caffeoylquinic acids compared to the control group cultivated at the same time was observed. The proportion of quercetin derivatives after 42 days was approximately the same in all treatments regardless of the CuSO_4_ treatment (Figure 4).

The predominant phenolic acids were caffeoylquinic acids. The main caffeoylquinic acid was chlorogenic acid in all tested treatments. The highest content of chlorogenic acid was quantified in plantlets grown for 42 days and treated with 3 mM CuSO_4_ (Table 3). There were statistically significant differences in chlorogenic acid content among most of the treatments tested. There is a noticeable trend of higher chlorogenic acid content in plantlets treated with a higher concentration of added CuSO_4_ for a longer cultivation time. The same applies to 4-caffeoylquinic acid, the second most abundant caffeoylquinic acid. The least represented caffeoylquinic acid in all experimental systems was *cis*-5-caffeoylquinic acid. Plantlets treated with 3 mM CuSO_4_ and grown for 42 days also contained the highest content of it, while plantlets treated with 0.5 mM CuSO_4_ grown for 21 days contained the lowest content of this compound. More detailed data on the content of individual caffeoylquinic acids in the stem node culture of *M. nigra* treated with different CuSO_4_ contents and cultivated for different periods of time can be found in Table 3. Among the *p*-coumaric acid derivatives, *p*-coumaric acid hexoside 1 was predominant in plant material cultivated under all tested conditions. The highest content of *p*-coumaric acid hexoside 1 was in the plantlets that were grown for 42 days and were treated with 3 mM CuSO_4_. There were statistical differences between almost all observed treatments. A trend of increasing content of *p*-coumaric acid hexoside 1 can be observed with the aging of the stem node culture and the higher concentration of added CuSO_4_. The second most represented *p*-coumaric acid derivative in most treatments (with the exception of plantlets grown for 21 days with the addition of 3 mM CuSO_4_) is *trans*-5-*p*-coumaroylquinic acid. The content of this compound also increases with a longer cultivation time and the addition of CuSO_4_ of a higher concentration. In most treatments, the third most represented phenolic acid from this group was *cis*-5-*p*-coumaroylquinic acid. The highest content of this compound was found in plantlets grown for 42 days with the addition of 3 mM CuSO_4_. Smaller amounts of *p*-coumaric acid hexoside 2 were identified in all treatments. Plantlets grown for 42 days in the control group contained the most of this compound. For this compound, it is not possible to describe a trend that would indicate a change in concentration in relation to the cultivation time and the concentration of the CuSO_4_ treatment. The same applies to 3-*p*-coumaroylquinic acid. Plantlets at day 0 of the experiment and grown for 21 days with the addition of 0.5 mM CuSO_4_ contained contents lower than the limit of quantification (LOQ). More detailed data on the content of individual derivatives of *p*-coumaric acid in the nodal culture of *M. nigra* treated with different contents of CuSO_4_ and cultivated for different periods of time can be found in Table 4.

Kaempferol acetyl-hexoside reached the highest content among the identified kaempferol derivatives in *M. nigra* stem node culture. Plantlets grown for a longer time and treated with higher concentrations of CuSO_4_ contained a higher content of it. The highest content was quantified in plantlets grown for 42 days and treated with 3 mM CuSO_4_. Its content differed statistically significantly between most of the tested treatments. Kaempferol dirhamnosyl-hexoside and kaempferol acetyl-rhamnosyl-hexoside followed among the determined kaempferol derivatives in terms of the highest determined content. The content of these two kaempferol derivatives also differed statistically between almost all tested conditions. They both reached higher content in plantlets which were grown for a longer time and treated with higher concentrations of CuSO_4_. Kaempferol rhamnosyl-hexoside had the lowest content among all identified kaempferol derivatives. The highest content was quantified in the plantlets sampled at day 0 of the experiment, and with a longer cultivation time and more concentrated CuSO_4_ treatment, its content decreased and differed statistically significantly among most experimental systems. Among the quercetin derivatives, the highest content was quantified for rutin. This was measured in plantlets cultivated for 42 days with an addition of the 3 mM CuSO_4_ solution. The rutin content differed statistically significantly among most of the tested treatments. A trend of increasing rutin content with a longer cultivation time and treatment with higher CuSO_4_ contents was observed. The same applied for quercetin derivatives, which followed rutin in terms of quantified content in the following order: quercetin malonyl-hexoside, quercetin acetyl-rhamnosyl-hexoside, quercetin rhamnosy-hexoside, quercetin acetyl-hexoside, and quercetin-3-glucoside. The lowest content among the quercetin derivatives was quantified for quercetin dirhamnosyl-hexoside. The contents of this quercetin derivative also differed statistically significantly between most of the experimental systems. Unlike other quercetin derivatives, the maximum amount of this derivative was determined at day 0 of the experiment, and with a longer cultivation time and an increasing concentration of CuSO_4_ treatment, its content decreased. More detailed data on the content of identified flavonoids in *M. nigra* nodal culture treated with different concentrations of CuSO_4_ and grown for different periods of time are presented in Table 5 and Table 6.

### 2.6. Effect of Treatment with CuSO_4_ and Cultivation Time on the Phenolic Compound Syntheses in M. nigra Stem Node Culture

The effect of CuSO_4_ treatment and cultivation time on the phenolic compound syntheses was further tested with a two-way analysis of variance. CuSO_4_ treatment, stem node culture cultivation time, and the combination of both had a significant effect on TPC and TFC. The content of most of the identified and quantified phenolic acids and flavonoids was also significantly influenced by treatment with CuSO_4_, cultivation time, and the combination of both. Treatment with CuSO_4_ has a significant effect on the content of all quantified individual phenolic compounds. Cultivation time has a significant influence on the content of all quantified phenolic compounds with the exception of *cis*-5-*p*-coumaroylquinic acid. Also, the combination of treatment with CuSO_4_ and cultivation time has a significant effect on the majority of quantified phenolic compounds in *M. nigra* nodal culture—the aforementioned combination only does not a significant effect on the content of *cis*-5-caffeoylquinic acid, *cis*-5-*p*-coumaroylquinic acid, and quercetin dirhamnosyl-hexoside. The results from a two-way analysis of variance are presented in Table S2.

In order to evaluate the influence of the CuSO_4_ concentration of the treatment and the influence of the length of cultivation on the biosynthesis of phenolic compounds, a comparison of the mean contents (Table 3, Table 4, Table 5 and Table 6) was performed. The trend was positive for TPC and TFC for the level of the CuSO_4_ treatment concentration and for the cultivation time. A strong positive trend was also found between the level of the CuSO_4_ treatment concentration and the content of *p*-coumaric acid hexoside 1, chlorogenic acid, 4-caffeoylquinic acid, *cis*-5-caffeoylquinic acid, kaempferol dirhamnosyl-hexoside, quercetin rhamnosyl-hexoside, rutin, quercetin-3-glucoside, quercetin acetyl-rhamnosyl-hexoside, kaempferol acetyl-rhamnosyl-hexoside, quercetin malonyl-hexoside, kaempferol acetyl-hexoside, and quercetin acetyl-hexoside. A negative trend was observed between the CuSO_4_ concentration and the quercetin dirhamnosyl-hexoside content. A positive trend was observed between the cultivation time and the content of *p*-coumaric acid hexoside 2, 3-*p*-coumaroylquinic acid, rutin, quercetin acetyl-rhamnosyl-hexoside, kaempferol acetyl-rhamnosyl-hexoside, quercetin acetyl-hexoside, chlorogenic acid, trans-5-*p*-coumaroylquinic acid, kaempferol dirhamnosyl-hexoside, quercetin rhamnosyl-hexoside, and quercetin-3-glucoside. A negative trend was found between the cultivation time and quercetin dirhamnosyl-hexoside content and kaempferol rhamnosyl-hexoside content.

### 2.7. Correlations Between Analyzed Phenolic Compounds

**Figure 5:**
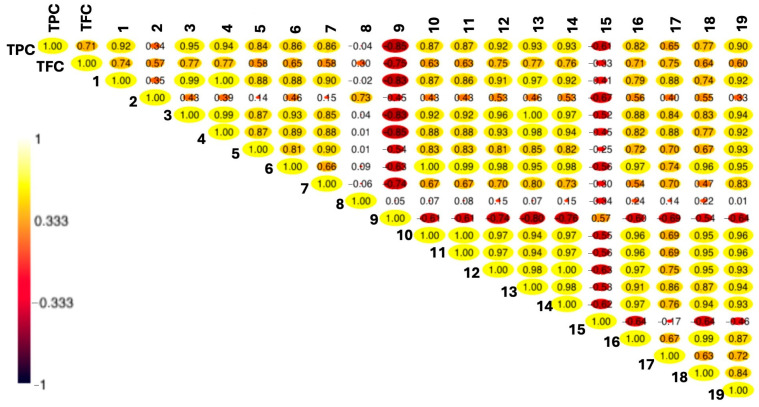
The Pearson correlation matrix for the relationships between total TPC, TFC, and individual phenolic compounds detected in *M. nigra* stem node culture samples treated with varying concentrations of CuSO_4_. The matrix displays correlation coefficients (r), with values ranging from −1 (strong negative correlation) to +1 (strong positive correlation), alongside a color gradient for visual interpretation: yellow represents positive correlations, while red indicates negative correlations. The ellipses further depict the strength and direction of the correlations, where narrower ellipses correspond to stronger relationships. (1) *p*-coumaric hexoside 1; (2) *p*-coumaric acid hexoside 2; (3) chlorogenic acid; (4) 4-caffeoylquinic acid; (5) *cis*-5-caffeoylquinic acid; (6) *trans*-5-*p*-coumaroylquinic acid; (7) *cis*-5-*p*-coumaroylquinic acid; (8) 3-*p*-coumaroylquinic acid; (9) quercetin dirhamnosyl-hexoside; (10) kaempferol dirhamnosyl-hexoside; (11) quercetin rhamnosyl-hexoside; (12) rutin; (13) quercetin-3-glucoside; (14) quercetin acetyl-rhamnosyl-hexoside; (15) kaempferol rhamnosyl-hexoside; (16) kaempferol acetyl-rhamnosyl-hexoside; (17) quercetin malonyl-hexoside; (18) quercetin acetyl-hexoside; (19) kaempferol acetyl-hexoside.

Pearson correlation coefficients were calculated to evaluate the relationships between various phenolic compounds. The correlations are presented in Figure 5, with values ranging from −1 to +1, where values closer to +1 or −1 indicate a stronger correlation. A strong positive correlation was found between TPC and TFC, indicating that these two parameters tend to increase or decrease in parallel. Similarly, a positive correlation was determined between the content of most of the quantified individual phenolic compounds. The content of the predominant representative of *p*-coumaric acids, *p*-coumaric acid hexoside 1, was negatively correlated only with 3-*p*-coumaroylquinic acid, quercetin dirhamnosyl-hexoside, and kaempferol rhamnosyl-hexoside. With the contents of the other quantified compounds, *p*-coumaric acid hexoside 1 correlated positively. Chlorogenic acid, the predominant caffeoylquinic acid, was negatively correlated with quercetin dirhamnosyl-hexoside and kaempferol rhamnosyl-hexoside, while with the contents of the other compounds, it showed positive correlations of varying strength. The same applied for the predominant representative of the quercetin derivatives, rutin, and for the main representative of the kaempferol derivatives, kaempferol acetyl-hexoside.

### 2.8. Influence of CuSO_4_ Treatment on the Antioxidant Activity of Acidified Methanolic Extracts of M. nigra Stem Node Culture

The results of the DPPH antioxidant assay of the acidified methanol extracts of *M. nigra* stem node culture are presented in Table 7. To more precisely assess the power of the antioxidant activity of the extracts, the antioxidant activity of the standards of individual phenolic compounds at a concentration of 0.001 M was also measured. Among the phenolic standards, the lowest antioxidant activity was shown by the 0.001 M solution of *p*-coumaric acid and the highest by the 0.001 M solution of rutin (2.29 times higher than *p*-coumaric acid). The antioxidant activity of the acidified methanol extracts of *M. nigra* nodal culture differed statistically significantly between most treatments, but not between all. The highest antioxidant activity was shown by the extract of samples grown for 42 days and treated with 3 mM CuSO_4_; however, it was 23.5 times lower than the antioxidant activity of 0.001 M *p*-coumaric acid, which showed the lowest antioxidant activity among the tested standards. The lowest antioxidant activity was shown by the extract of the control sample cultivated for 21 days. Compared to the extract of the sample, which showed the highest antioxidant activity, it was 3.83 times lower. Compared to the 0.001 M standard solution of *p*-coumaric acid, it was 90 times lower. The antioxidant activity of the extracts in samples treated with CuSO_4_ of all tested concentrations increased throughout the experiment. A slightly different trend in changing antioxidant activity was determined for the extracts of plantlets grown under control conditions. After 21 days of the experiment, their antioxidant activity decreased compared to day 0 of the experiment, and after 42 days of the experiment, it was higher than at day 0 of the experiment.

### 2.9. Correlations Between Total Phenolic Content, Total Flavonoids Content, and Phenolic Compound Group Contents in Relation to Antioxidant Activity of Extracts

Pearson’s correlation coefficient was calculated to determine the connections between the TPC, TFC, and quantified content of the groups of phenolic compounds in relation to the DPPH antioxidant activity of extracts (Table 8). TPC and TFC both strongly positively correlated with the antioxidant activity of the extracts. The contents of *p*-coumaric acid derivatives (0.941 **), caffeoylquinic acids (0.960 **), quercetin derivatives (0.918 **), and kaempferol derivatives (0.918 **) also strongly positively correlated with the antioxidant activity of the extracts. By comparing the average antioxidant activity values of plantlets grown under different conditions (Table 7), we also detected a positive trend between the higher antioxidant activity and higher CuSO_4_ concentration of the treatment. Also, a positive trend between the higher antioxidant activity of plantlets and longer cultivation time was observed.

### 2.10. Influence of CuSO_4_ Treatment on the Yield of Individual Phenolic Compounds in Stem Node Culture of M. nigra

Yields of quantified individual phenolic compounds per 1 L of MS medium obtained from an *M. nigra* stem node culture exposed to CuSO_4_ elicitation were calculated based on the data of the dry weight and the contents of quantified individual phenolic compounds. The yields of all quantified phenolic compounds differed statistically significantly among most of the tested experimental systems (Tables S3–S6). The highest yield among all individual phenolic compounds was that of chlorogenic acid (21.3362 mg/L of MS medium) after 42 days of cultivation and treatment with 3 mM CuSO_4_. The highest yield of most quantified phenolic compounds was also obtained after 42 days of treatment of nodal culture with 3 mM CuSO_4_ (Table S3). The highest yield under these conditions was also observed for *p*-coumaric acid hexoside 1, 4-caffeoylquinic acid, *cis*-5-caffeoylquinic acid, *trans*-5-*p*-coumaroylquinic acid, *cis*-5-*p*-coumaroylquinic acid, kaempferol dirhamnosyl-hexoside, rutin, quercetin-3-glucoside, quercetin acetyl-rhamnosyl-hexoside, kaempferol acetyl-rhamnosyl-hexoside, quercetin malonyl-hexoside, quercetin acetyl-hexoside, and kaempferol acetyl-hexoside. A different trend was observed for quercetin rhamnosyl-hexoside, which had the highest yield after 42 days under the 1 mM CuSO_4_ treatment. In contrast, *p*-coumaric acid hexoside 2, 3-*p*-coumaroylquinic acid, and quercetin dirhamnosyl-hexoside had the highest yields after 42 days of cultivation under control conditions. The highest yield for kaempferol rhamnosyl-hexoside was obtained after 21 days of cultivation under control conditions. Among the *p*-coumaric acid derivatives, the highest yield was achieved by *p*-coumaric acid hexoside 1, among the kaempferol derivatives by kaempferol acetyl-hexoside, and among the quercetin derivatives by rutin. More detailed data on the yields of caffeoylquinic acids (Table S3), *p*-coumaric acid derivatives (Table S4), kaempferol derivatives (Table S5), and quercetin derivatives (Table S6) can be found in the Supplementary Tables.

## 3. Discussion

### 3.1. The Influence of CuSO_4_ Treatment on Plantlets Development

Based on morphological signs, we can conclude that the plantlets grown in the control group and those treated with 0.5 mM CuSO_4_ grew best. They were the largest in size, and there was no visible chlorosis, necrosis, or deformation on their leaves. For plantlets that are genetically identical and at the same time larger, we can conclude that they were exposed to less stress. Environmental stresses decrease plantlets’ growth [48]. Chlorophyll gives leaves their characteristic green color [49]. Its decrease in leaves is associated with exposure to stress [50]. Given the fact that the plantlets in the control group and plantlets treated with 0.5 mM CuSO_4_ grew visibly throughout the experiment, and that there were no changes in leaf color, we can assume that they were not exposed to stress and that these were suitable growing conditions for them. However, without performing analyses of chlorophyll content, we cannot completely state that the chlorophyll content was the same throughout the experiment. Plantlets treated with 1 mM CuSO_4_ and 3 mM CuSO_4_ were visibly smaller, and both had the presence of marginal chlorosis. Plantlets treated with 3 mM CuSO_4_ were the smallest. This is due to the fact that excess copper inhibits growth and disrupts important cellular processes [18]. More marginal chlorosis was also observed on the leaves of plantlets treated with 3 mM CuSO_4_. More marginal chlorosis on plantlets treated with higher concentrations of copper was also observed in previous studies, e.g., in the tissue culture of *Sideritis raeseri* [51], the tissue culture of *Philodendron selloum* [52], and in the tissue culture *of Stevia rebaudiana* [53]. A common and major symptom of plant Cu toxicity is leaf marginal chlorosis, which results in yellow/white spots or lesions [54]. Cu has been shown to increase susceptibility to photoinhibition in vitro using isolated thylakoids or photosystem II complexes. Excess Cu-induced susceptibility to photoinhibition is particularly severe in intact leaves. A reduction in chlorophyll concentration has been observed to accompany Cu excess concomitant with ultrastructural changes in chloroplasts, such as a reduction in thylakoid membranes. Excess Cu interferes with the biosynthesis of the photosynthetic machinery and modifies the pigment and protein components of photosynthetic membranes [54,55]. In future studies, it would be necessary to more precisely determine the threshold concentration of CuSO_4_ treatment that does not cause negative effects on *M. nigra* stem node culture development, while still promoting an increase in phenolic content.

### 3.2. The Influence of CuSO_4_ Treatment on Fresh and Dry Weights of Plantlets

Plantlets grown under all treatments gained fresh and dry weight throughout the experiment. The highest fresh and dry weight was gained by plantlets treated with 0.5 mM CuSO_4_, while plantlets treated with 3 mM CuSO_4_ had the lowest weight. Cu plays a vital role in the proper growth and development of plants. It is involved in many morphological, physiological, and biochemical processes [56]. However, Cu excess reduced plant biomass gains [57]. A high dose of Cu is known to damage protein synthesis and enzyme activity and alter membrane permeability, inducing the inhibition of photosynthesis and respiration. Consequently, the ability to gain biomass is reduced [58]. Based on this, we conclude that the concentration of Cu in the stem node culture treated with 0.5 mM CuSO_4_ had a priming effect, while the treatments with 1 mM and 3 mM CuSO_4_ were toxic. A similar trend in the biomass production of various plants treated with CuSO_4_ was also found in other previous studies. Plantlets to which lower concentrations of CuSO_4_ were added gained more biomass compared to the control group, while plantlets to which higher concentrations of CuSO_4_ were added gained less biomass. This, for example, applies to the callus culture of *Phoenix dactylifera* tissue culture [59], *Silybum marianum* cell culture [60], and banana tissue culture [61]. The concentrations of CuSO_4_ treatment, which has a toxic effect on the plant and consequently reduces the efficiency of biomass production, are not comparable. This is due to the range between priming and toxic concentration for plant species significantly differing, and accurate estimation is required when applying Cu to plants in regard to genotype dependent tolerance [62]. In future studies, it would be necessary to examine the effect of several different concentrations of CuSO_4_ on biomass production in *M. nigra* stem node cultures in order to more accurately determine the highest concentration of CuSO_4_ that does not inhibit biomass production. This would be important because increased biomass production would yield active compounds, much needed by the pharmaceutical and nutraceutical industries [63]. It would also be suitable to determine biomass more frequently in order to determine the time period in which mass production is optimal. Biomass production was slower between the 21st and 42nd day than during the first 21 days of the experiment. This could be due to nutrient deficiency that causes a decline in plantlets mass gain [64] and occurs at the age when the plantlet consumes the necessary macro- and micronutrients from the culture medium [65].

### 3.3. The Influence of CuSO_4_ Treatment on Total Phenolic Content and Total Flavonoid Content in M. nigra Stem Node Culture

The highest TPC was determined in *M. nigra* stem node cultures treated with CuSO_4_ for 42 days. To the best of our knowledge, this is the first study to determine TPC in *M. nigra* nodal culture; therefore, the results (neither control) cannot be directly compared with previous studies. The extracts of *M. nigra* stem node culture sampled at the beginning of the experiment (day0) of the present experiment contained higher TPC than the hydromethanolic extracts of black mulberry leaves in the studies of Polumackanycz et al. [41], Chen et al. [66], and Kolayli et al. [67]. On the other hand, stem node cultures from the present study contained less TPC than *M. nigra* leaves in the studies of Zeni et al., Dalmagaro et al., and Jelen and Urbanek Krajnc [44,68,69]. In the study by Thabti et al., the TPC value in the ethanolic extracts of *M. nigra* leaves was comparable to the TPC value of acidified methanolic extracts of *M. nigra* nodal culture [70]. However, we cannot directly compare the TPC determined in the stem node cultures and in the leaves of plants grown in the natural environment [71]. It is also difficult to compare the TPC value objectively between studies, because the extraction procedures differ between studies [72,73]. Compared to different in vitro cultures of other plant species, the TPC values in stem node cultures of *M. nigra* were different. *Artemesia absinthum* callus culture elicited with thidiazuron [74], *Gloriosa superba* tissue culture [75], various blueberry tissue culture cultivars [76], *Argania spinosa* callus culture [77], and *Echinacea purpurea* culture elicited with various biotic elicitors [78] contained lower TPC than *M. nigra* nodal culture. On the other hand, the leaf culture of *Crataegus azarolus* elicited with additives of 2,4-dichlorophenoxyacetic acid and 6-benzylaminopurine [79] and *Givotia moluccana* tissue culture elicited with salicylic acid contained higher TPC than *M. nigra* nodal culture elicited with CuSO_4_ [80]. Phenolic compound syntheses in *M. nigra* nodal culture could be improved by adding biosynthetic precursors to the MS medium or by using other elicitors [81]. In future studies, the synthesis of phenolic compounds could be elicited with jasmonic acid [82], salycilic acid [82], methyl jasmonate [83], chitosan [84], yeast extract [84], NaCl [85], nanoparticles [86], exposure to lights of different wavelengths [87], and the addition of various heavy metals [88]. It would also be worthwhile to test the effectiveness of the combined use of different elicitors on the synthesis of phenolic compounds, as based on the previous studies, the combined application of different elicitors showed an improved accumulation of secondary metabolites due to their synergistic effect [89]. In the present study, elicitation with CuSO_4_ gave positive results as the highest TPC values were found in plantlets treated with the highest tested concentration of CuSO_4_ for the longest time. The results are in agreement with other studies, as the addition of Cu also stimulated the synthesis of phenolic compounds in the root culture of *Althea officinalis* [25], the root suspension culture of *Panax ginseng* [90], and in the cell suspension culture of *Celastrus paniculatus* [91]. Authors demonstrated that copper stress leads to the activation of the phenylpropanoid pathway by stimulating the phenylpropanoid biosynthetic pathway in plants by up-regulating the activities of key biosynthetic enzymes like PAL, shikimate dehydrogenase (SKDH), glucose-6-phosphate dehydrogenase (G6PDH), and cinnamyl alcohol dehydrogenase (CADH). Additionally, the enzyme PPO helps during the process of reactive oxygen species (ROS) scavenging and enhances a plant’s resistance to excess Cu stress conditions [17,92]. For a better understanding and more effective optimization of CuSO_4_ elicitation of phenolic compounds, it would be necessary to study the effect of the CuSO_4_ treatment on the activity of the most important enzymes of the phenylpropanoid metabolic pathway in the future.

The TFC values in the stem node cultures of *M. nigra* grown under control conditions in the present study are comparable or slightly higher than those in a previous study conducted by Abd El-Mawla et al. [93]. In the present study, the highest TFC was observed in nodal culture samples treated for the longest tested time with the highest tested concentration of CuSO_4_. Compared to other plant cultures, the TFC in CuSO_4_-elicited stem node cultures of *M. nigra* is average, as in vitro cultures of some species contain less total flavonoids and some more. The callus culture of *Passiflora quadrangularis* contained less total flavonoids than the stem node culture of *M. nigra* [94]. The in vitro culture of *Momordica charantia* [95] and the differently treated root suspension cultures of *Panax ginseng* [90] contained comparable TFC values to the stem node culture of *M. nigra*. The callus culture of *Sophora flavescens* [96] and the cell culture of *Astragalus missouriensis* [97] contained higher flavonoid content than the nodal culture of *M. nigra.* In the present study, the highest TFC values were achieved by samples elicited with CuSO_4_ for 42 days, indicating that CuSO_4_ is a successful elicitor of flavonoid biosynthesis in *M. nigra* stem node culture. Treatment with CuSO_4_ also increased the content of total flavonoids in the callus culture of *Orthosiphon stamineus* [98], in the cell culture of *Digitalis lanata* [99], and in the root suspension culture of *Panax ginseng* [90]. The increased activity of enzymes such as PAL, trans-cinnamate 4-monooxygenase (C4H), and 4-coumarate-CoA ligase (4CL) affects the synthesis of flavonoids as well as other phenolic compounds. The action of the CHS enzyme represents the beginning of the specific flavonoid pathway [100]. The CHS enzyme which catalyzes the committal step in flavonoid biosynthesis is also up-regulated by copper, resulting in a higher content of total flavonoids [101].

In future studies, it would be suitable to test the influence of different elicitors for flavonoid synthesis in *M. nigra* nodal culture. It would be particularly worthwhile to test elicitation with various nanoparticles [102], methyl jasmonate [103], salinity stress [104], and salicylic acid [105] for flavonoid synthesis.

The content of total phenolic compounds and total flavonoids also depends on the extraction solvent used, because the nature of the solvent and its polarity significantly impacted the phenolic extraction [106,107,108]. Therefore, it would be necessary to investigate which extraction solvent is most effective in extracting phenolic compounds from *M. nigra* plant material cultivated in stem node culture. It would also be suitable to examine the effectiveness of deep eutectic solvents due to their environmentally friendly characteristics [109].

Future research should also investigate the effect of a wider range of concentrations of CuSO_4_ in order to optimize the synthesis of phenolic compounds. There is also a need to more accurately assess how the content of phenolic compounds in *M. nigra* stem node culture changes during the cultivation period. For further optimization of the stem node culture of *M. nigra* for the production of phenolic compounds, it would be suitable to conduct an experiment with several different genotypes. The content of phenolic compounds depends on the genotype [110], and different genotypes have different resistances to heavy metal stress [111].

In addition to phenolic compounds, *M. nigra* leaves contain various terpenoids [112], carotenoids [68], and the alkaloid 1-deoxynojirimycin [113]. Therefore, it would be suitable to identify and quantify these compounds in combination with CuSO_4_ treatment, because the co-production of metabolites has economic and operational advantages [114].

Future research should explore various in vitro cultures of *M. nigra* to enhance phenolic compound production [115]. Establishing hairy root cultures could further utilize its bioactive root compounds [116], while investigating in vitro fruiting may provide insights into its fruit’s bioactive potential [117,118].

In the present study, we found that *M. nigra* accumulates higher levels of phenolic compounds under copper stress. A higher accumulation of phenolic compounds indicates that the plant is more tolerant to heavy metal stress [119]. Therefore, it would be worthwhile to conduct future research on the potential of *M. nigra* as a copper phytoremediator. Such research would be important from the perspective that the Cu contamination of vineyards is a major environmental problem [120].

### 3.4. Qualitative and Quantitative Analyses of Phenolic Compounds in Stem Node Culture of M. nigra

To the best of our knowledge, this is the first study to identify the phenolic profile of *M. nigra* stem node culture. Nineteen phenolic acids and flavonoids were identified, which can be classified as caffeoylquinic acids, *p*-coumaric acid derivatives, kaempferol derivatives, and quercetin derivatives. Chlorogenic acid was the dominant caffeoylquinic acid, *p*-coumaric acid hexoside 1 was the dominant *p*-coumaric acid derivative, kaempferol acetyl-hexoside was the dominant kaempferol derivative, and rutin was the dominant quercetin derivative. A similar phenolic profile has been identified in *M. nigra* leaves in previous studies [30,36,69,121,122,123]. In some of the aforementioned studies, protocatechuic acid, caffeic acid, cinnamic acid, ferulic acid, sinapic acid, catechin, naringenin, syringic acid, gallic acid, vanillic acid, luteolin, ellagic acid, and epicatechin were also determined [30,36,71,121,124,125]. The phenolic profiles of in vitro cultures and wild plants are not comparable, even though they are plants of the same species, due to the different environmental conditions of in vivo and in vitro grown plants and the developmental stage [126]. It is also important to consider the genotype-specific differences in the phenolic profile [127]. Also, in some previous research, only HPLC-PDA and not HPLC-MS were used to identify phenolic compounds. PDA detection is not sufficient to discriminate between compounds with similar spectroscopic characteristics, and MS is needed to define the compounds on their molecular masses and to exclude the possibility of interferences [128].

Cu stress alters the ratios of the contents of individual phenolic compounds [129]. The mean total content of the four determined groups of phenolic compounds increased upon the stem node culture of *M. nigra* elicitation with CuSO_4_. However, the ratio between the concentrations of the groups of phenolic compounds changed upon elicitation with CuSO_4_. An increased concentration of CuSO_4_ treatment significantly increased the proportion of kaempferol derivatives. A longer exposure time to CuSO_4_ further increased the proportion of kaempferol derivatives. This increase in the concentration of kaempferol derivatives is probably related to its ability to chelate Cu. The BCS test in the study by Riha et al. proved that kaempferol chelates Cu better than some other flavonoids [130]. The content of quercetin derivatives increases mainly with a longer exposure time to CuSO_4_ and not so much with the content of added CuSO_4_. This increase in the content of quercetin derivatives with the cultivation time could be related to the aging of the in vitro culture, because ROS are generated during aging [131], which induces the biosynthesis of quercetin and other flavonoids in plantlets [132]. With increasing concentration of CuSO_4_ treatment, the proportion of *p*-coumaric acid derivatives increased. This is most likely related to the increase in the proportion of kaempferol and quercetin derivatives. *p*-coumaric acid plays a central role in secondary metabolism because it is a precursor for flavonoids biosynthesis. It can be subsequently transformed to flavonoids, and also into kaempferol and quercetin derivatives [122]. The absolute content of caffeoylquinic acids increased with the aging of the culture and a higher concentration of CuSO_4_ treatment. However, its proportion decreased compared to other groups of phenolic compounds. This is most likely due to the fact that the heavy metal chelating ability and the antioxidant activity of caffeoylquinic acids are lower than those of flavonoids—consequently, under stress conditions, the biosynthesis of flavonoids was more intense than the biosynthesis of caffeoylquinic acids [123].

The predominant derivatives of *p*-coumaric acid were *p*-coumaric acid hexoside 1 and *trans*-5-*p*-coumaroylquinic acid. Both of them reached their highest levels in stem node culture treated with 3 mM CuSO_4_ for 42 days. This is probably due to the fact that heavy metal stress increases the activity of PAL. PAL can catalyze the nonoxidative elimination of ammonia from L-phenylalanine to give trans-cinnamic acid. Following PAL activity, the hydroxylation catalyzed by C4H gives rise to *p*-coumaric acid and its derivatives [133]. *p*-coumaric acid derivatives exhibit various bioactivities, including antioxidant, anti-inflammatory, antimutagenic, anti-ulcer, antiplatelet, and anti-cancer activities, [122]. The content of *p*-coumaric acid hexoside in a cell culture of *Vitis amurensis* elicited with the addition of phenolic acids is lower than in a nodal culture of *M. nigra* elicited with CuSO_4_ [134]. *Cydonia oblonga* callus culture contained less *p*-coumaroylquinic acid [135], *Hypericum perforatum* callus culture contained comparable content [136], whereas *Scorzonera radiata* callus culture contained higher *p*-coumaroylquinic acid than *M. nigra* stem node culture elicited with CuSO_4_ [137].By reviewing the current literature available in the field, it can be found that the in vitro cultures of certain plant species contain lower contents and some higher contents of *p*-coumaric acid derivatives. Therefore, additional studies would be necessary to establish a more efficient production of *p*-coumaric acid derivatives. It would be suitable to optimize the synthesis of *p*-coumaric acid derivatives by testing several different CuSO_4_ treatments, cultivation times, and other elicitors successful in previous studies [82,83,84,85,86,87,88].

The predominant caffeoylquinic acids in *M. nigra* stem node culture were chlorogenic acid and 4-caffeoylquinic acid. Both of them reached the highest levels in cultures elicited with 3 mM CuSO_4_ for 42 days. It was previously reported that the biosynthesis of caffeoylquinic acids is induced by heavy metal stress [138]. The C3H gene influences the synthesis of phenolic acids, and its up-regulation increases levels of caffeoylquinic acid derivatives [139,140]. These compounds are of interest in various fields because they exhibit antioxidant, antibacterial, anti-cancer-related, antiviral, anti-Alzheimer, and neuroprotective activity [141]. The stem node culture of *M. nigra* elicited with CuSO_4_ could potentially be applied for the production of chlorogenic acid. The chlorogenic acid content in the in vitro culture of *Eryngium planum* cultivated with different medium supplements [142], the in vitro culture of *Schisandra chinensis* treated with different growth regulators [143], the callus culture of *Varthemia persica* elicited with different medium supplements [144], and the tissue culture of *Berula erecta* elicited with blue light and low temperature [87] was lower than in the nodal culture of *M. nigra* elicited with CuSO_4_. Even the leaf culture of *Cynara cardunculus* [145], which is described as a species suitable for the production of chlorogenic acid [115], contained lower chlorogenic acid contents. Moreover, the content of 4-caffeoylquinic acid in *Gardenia jasminoides* cell culture elicited with salicylic acid or methyl jasmonate [146], the callus culture of *Rumex thyrsiflorus* elicited with NaCl [147], and the shoot culture of *Aronia melanocarpa* grown on MS medium of different compositions was lower than in the nodal culture of *M. nigra* [148]. Based on the above studies, it can be concluded that the stem node culture of *M. nigra* elicited with CuSO_4_ shows potential for the in vitro production of caffeoylquinic acids. Therefore, it would be suitable to further optimize the elicitation with CuSO_4_ and to test the efficiency of large-scale production [149].

The predominant quercetin derivatives were rutin and quercetin malonyl-hexoside. Both reached their highest levels after 42 days of treatment with 3 mM CuSO_4_. It was previously reported that CuSO_4_ stress enhances quercetin synthesis [150]. Exposure to heavy metals in plants increases the activity of the F3′H gene that is responsible for the synthesis of dihydroquercetin, a precursor in the synthesis of quercetin [151,152,153]. Quercetin is presumed to have antioxidant, anti-inflammatory, immunoprotective, and anticarcinogenic effects [154]. The callus culture of caper elicited with polyamines contained lower rutin content [155], while the callus culture of *Saussurea involucrata* [156] contained comparable rutin content to the stem node culture of *M. nigra* elicited with CuSO_4_. The rutin content in the root callus culture of *Rumex hastatus* elicited with various elicitors [157], in different clones of the hairy root culture of *Fagopyrum esculentum* [158], and in the in vitro culture of *Ruta graveolens* elicited with sorbitol [159] is higher than in the elicited nodal culture of *M. nigra*. Data on the content of quercetin malonyl-hexosides in in vitro cultures are limited. The content of quercetin malonyl-glycoside in the tissue cultures of lettuce cultivar ‘RutgersScarlet’ [160] is higher than in the stem node culture of *M. nigra* elicited with CuSO_4_. Based on the above discussed comparison with other articles, we can conclude that the nodal culture of *M. nigra* elicited with CuSO_4_ is not suitable for the production of quercetin derivatives. Future studies should primarily examine the influence of elicitors that have successfully induced the biosynthesis of quercetin in the in vitro cultures of other plant species, e.g., methyl jasmonate [161], sucrose [162], AgNO_3_ [163], polyamines [155], and *Bacillus* QV15 [164].

The dominant quantified kaempferol derivatives were kaempferol acetyl-hexoside and kaempferol dirhamnosyl-hexoside. Both reached their highest levels in cultures elicited with 3 mM CuSO_4_ for 42 days. In the biosynthesis of kaempferol, dihydrokaempferol is converted into kaempferol via the FLS gene [165]. Heavy metals up-regulate the FLS gene [166]. Kaempferol is safe and nontoxic, as well as exhibits anti-inflammatory, anti-cancer, and anti-diabetes effects [167]. Kaempferol acetyl-hexoside is a rare flavonoid acetylated glucoside [168]. To the best of our knowledge, there is no study examining the content of kaempferol acetyl-hexoside in plant in vitro cultures; therefore, the content determined in the present study cannot be compared. We also did not find data on the content of kaempferol dirhamnosyl-hexoside in in vitro cultures. Future studies should be aimed at quantifying these two kaempferol derivatives in the in vitro cultures of other plant species and comparing their content with the content in the nodal culture of *M. nigra* elicited with CuSO_4_—only in this way could we assess the suitability of the stem node culture of *M. nigra* for their production. It would also be suitable to test the influence of elicitors, which have previously shown efficiency for kaempferol derivatives syntheses in the in vitro cultures of other plant species, e.g., jasmonic acid [169], kinetin [170], yeast extract [170], salicylic acid [171], abscisic acid foliar application [172], methyl jasmonate [173], iron chelate [174], and sodium metasilicate [174].

### 3.5. Effect of Treatment with CuSO_4_ and Cultivation Time on the Phenolic Compound Syntheses in M. nigra Stem Node Culture

Two-way analysis of variance showed that the contents of total phenolics, total flavonoids, and most quantified phenolic compounds were significantly influenced by the copper treatment, cultivation time, and combination of both. The further comparison of the average contents of quantified compounds showed a positive trend between TPC, TFC, and the content of most individual phenolic compounds with the higher concentration of CuSO_4_ treatment and with the longer cultivation time. The influence of the concentration of Cu treatment on the TPC, TFC, and on the content of most quantified individual phenolic compounds is probably related to the gradual increase in PAL activity by the addition of Cu [175]. PAL modulates the synthesis of phenolic compounds and catalyzes the conversion of phenylalanine to cinnamic acid, the primary mediator in phenolics biosynthesis and the precursor of various phenylpropanoids [176]. To better understand and optimize the effect of CuSO_4_ elicitation on PAL activity in *M. nigra* stem node culture, future research should determine the effect of CuSO_4_ on PAL activity and the effect of CuSO_4_ on the expression of PAL-related genes. However, the mean content of a small number of individual phenolic compounds did not show any trend or even showed a negative trend with the concentration of CuSO_4_ treatment. This is probably related to the fact that the compounds whose content decreased have functions which are not related to defense against Cu stress. Different phenolic compounds have different roles in plants [3,177]. Quercetin dirhamnosyl-hexoside is the only individual phenolic compound which showed a strongly negative trend with the concentration of added CuSO_4_. The exact role of quercetin dirhamnosyl-hexoside in plants is unknown, but its difference compared to other quercetin derivatives is shown in the study by Šelih et al. [33] on *M. alba* growing in the natural environment. In the aforementioned study, this was the only quercetin derivative whose content was significantly affected by the pruning practice. Currently, we do not have enough data to draw conclusions about why CuSO_4_ treatment causes a decrease in the content of some metabolites.

The mean values of TPC and TFC, and the mean contents of most quantified individual phenolic compounds, showed positive trends with the longer cultivation time. An increase in the production of phenolic compounds with culture age is probably associated with a decrease in growth and a decline in protein synthesis [178,179]. However, the cultivation time did not show a trend with the content of certain phenolic compounds, or, it was negative, because different phenolic compounds followed different patterns during plant development and aging [180]. To better understand the influence of age on the content of particular phenolic compounds in *M. nigra* stem node culture, additional research would be needed to study changes in the activity of key enzymes for the synthesis of phenolic compounds, as well as monitoring changes in the expression of genes that regulate the synthesis of phenolic compounds.

### 3.6. Correlations Between Analyzed Phenolic Compounds

A strong positive correlation was determined between TPC, TFC and polyphenolic compounds [181]. This could be related to the fact that hydroxycinnamic acids can also act as precursors of flavonoids. The beginning of the synthesis of hydroxcinnamic acids and flavonoids is regulated by the same enzymes PAL, C4H, and 4CL. The first reaction for the synthesis of flavonoids is regulated after this stage with the CHS enzyme [182]. Based on this, we can conclude that under conditions where the respective initial enzymes are more active, more independent hydroxycinnamic acids are biosynthesized that may enter as precursors in the conversion into flavonoids. The only two identified and quantified phenolic compounds whose content shows a strong negative correlation with the content of almost all phenolics are quercetin dirhamnosyl-hexoside and kaempferol rhamnosyl-hexoside. This could be related to the fact that the accumulation of certain flavonoids can inhibit certain enzyme activity and suppress the synthesis of other flavonoids [183]. The reduced content of quercetin dirhamnosyl-hexoside and kaempferol rhamnosyl-hexosid compounds could be associated with the suppression of the FGRT gene, which regulates the synthesis of both aforementioned compounds. The FGRT gene is one of the key genes responsible for the resource-specific distribution of *O*-rhamnosylated flavonols and malonylated flavonol glycosides [184]. It is possible that these two glycosylated flavonoids are used as a glycosyl donors in the trans-glycosylation process [185]. In a previous study on *M. alba*, it was observed that quercetin dirhamnosyl-hexoside did not correlate with TPC, while other quercetin derivatives (quercetin malonyl-hexoside and rutin) showed a positive correlation [33]. However, to accurately understand the relationship between the biosynthesis of individual phenolics in the stem node cultures of *Morus nigra*, future studies should examine the enzymatic activity and gene expression involved in the phenylpropanoid metabolic pathway.

### 3.7. Influence of CuSO_4_ Treatment on the Antioxidant Activity of M. nigra Stem Node Culture and Correlations between CuSO_4_ Treatment, Cultivation Time, Total Phenolic Content, Total Flavonoid Content, and Phenolic Derivatives Content

The highest antioxidant activity measured by DPPH assay was found in extracts of plantlets treated with 3 mM CuSO_4_ for 42 days. This is comparable to some previous studies in which copper treatment also increased the antioxidant activity of plantlets extracts. CuSO_4_ treatment increased the antioxidant activity of the extract of the hairy root suspension culture of *Panax ginseng* [90], the extract of an in vitro cultivated tomato cultivar [186], and the extract of the in vitro culture of *Cicer spiroceras* [187]. In the present study, a strong positive correlation between antioxidant activity and TPC and TFC was determined. Such a positive trend has been previously determined in grape wastes [188], in brown rice [189], and in the callus culture of *Polyathia bullata* [190]. Phenolic compounds are effective scavengers of DPPH reagent [191]. This was also demonstrated in the present study, where all five of the used commercially available phenolic standards were much more effective scavengers of DPPH reagent than plant extracts (Table 7). The increase in antioxidant activity in plant extracts treated with higher concentrations of Cu for a longer time is probably influenced by the fact that not only the total mean contents of all phenolic groups increased but also the relative proportion of flavonoids increased, while the relative proportion of phenolic acids decreased (Figure 4). It was experimentally proved that the commercially available flavonoid standards of the same concentration exhibit better DPPH scavenging activity than the commercially available standards of phenolic acids (Table 6). However, *M. nigra* leaves, in addition to phenolic compounds, contain many other compounds, including betulinic acid [112], β-sitosterol [112], ascorbic acid [69], β-carotene [39], and 1-deoxynojirimycin [113]. All these compounds show the effective scavenging ability of DPPH reagent [192,193,194,195,196]. Consequently, the antioxidant activity of the extracts is not only influenced by the phenolic compounds but also by other compounds. In future studies, it would therefore be suitable to identify and quantify the terpenes, carotenoids, and alkaloids contained in *M. nigra* stem node culture and the effect of CuSO_4_ elicitation on their biosynthesis in order to better understand the antioxidant activity of *M. nigra* nodal culture extracts. Compared to the extracts of other plant species grown in vitro in previous studies, the antioxidant activity of *M. nigra* extracts elicited with CuSO_4_ is high. Lower antioxidant activity than *M. nigra* extracts elicited with CuSO_4_ was shown by the extracts of the elicited and non-elicited culture of *Bacopa monnieri* [197], the extracts of the callus culture of *Rania echinocarpa* [198], the light-elicited callus culture of *Stevia rebaudiana* [199], and in the callus culture of *Phytolacca americana* [200]. The antioxidant activity of extracts in the plant tissue culture of *Berula erecta* elicited with a combination of blue light and low temperature was comparable to the extract of the elicited nodal culture of *M. nigra* [87], whereas the extracts of *Cartaegous azarolus* leaf culture elicited with a combination of 2,4-dichlorophenoxyacetic acid and 6-benzylaminopurine showed higher antioxidant activity [79]. However, the evaluation of plant bioactivity is complicated; therefore, it is necessary to perform an appropriate combination of several different antioxidant assays [201]. In future studies, it would be suitable to examine the antioxidant activity of *M. nigra* extract with other antioxidant assays such as FRAP, ABTS, CUPRAC, RP, TRAP, TOSC, and ORAC assay [202].

### 3.8. Influence of CuSO_4_ Treatment on the Yield of Individual Phenolic Compounds in the Stem Node Culture of M. nigra

The stem node culture of *Morus nigra* elicited with CuSO_4_ yields the highest levels of caffeoylquinic acids, specifically chlorogenic acid and 4-caffeoylquinic acid. Therefore, it is the most suitable approach for producing these compounds. Many phenolic compounds are still obtained from soil-grown plants. However, soil-grown plants are not an ideal source of bioactive natural compounds due to slow plant growth, endangered plant species, and low in vivo productivity [203]. The chlorogenic acid content in the leaves of soil-grown plants is lower in some studies [41] and higher in others [30,44], while the 4-caffeoylquinic acid content is lower [36,44] than in the elicited nodal culture of *M. nigra*. The advantages of in vitro production are also independence from the season and an aseptic environment. *M. nigra* is a deciduous tree, so leaves can only be obtained seasonally [44]. Of the pests, it is subject to *Pseudaulacaspis pentagona* [204] and fungal infections with *Cercospora* spp [205]. Other advantages of the production of phenolic compounds with in vitro cultures are reliable, simple, and predictable production, more efficient extraction, interfering compounds for the synthesis of desired compounds can be removed by using biotechnology tools, higher production is possible, and various elicitation techniques can be tested and used [206]. For the efficient production of secondary metabolites by in vitro culture, it is necessary to optimize the accumulation of the biomass of the in vitro culture and the biosynthesis of the selected metabolite [12]. The yield of chlorogenic acid in plant in vitro cultures of different plant species is in some lower and in some higher than in the nodal culture of *M. nigra* elicited with CuSO_4_. The yield of chlorogenic acid in the cell culture of *Cecropia obtusifolia* [207] is lower than in the stem node culture of *M. nigra* elicited with CuSO_4_. However, the tissue culture of *Berula erecta* [87], the hairy root cultures of *Stevia rebaudiana* [208], and an in vitro culture of *Gardenia jasminoides* [146] have higher yields of chlorogenic acid. Data on the yields of 4-caffeoylquinic acid in plant in vitro cultures are scarce. The *Ageratina adenophora* culture had a lower yield of 4-caffeoylquinic acid [209]. It would be worthwhile to test the influence of elicitors that have been shown to stimulate the biosynthesis of caffeoylquinic acids in previous studies on other plant species [82,83,84,85,86,87,88]. For more efficient optimization, it would be necessary to study the biomass accumulation and the biosynthesis of desired caffeoylquinic acids separately [210]. However, the production of phenolic compounds with in vitro cultures also has disadvantages such as manual labor, the genetic stability of plants, and relatively high costs [211]. Due to the development of metabolic engineering, phenolic compounds can also be obtained through microbial production and fermentation [212,213]. The production of chlorogenic acid by the fermentation of coffee pulp has a lower yield [214], while the production methods with the genetically transformed fungus *Saccharomyces cerevisiae* [215] and the genetically modified bacterium *Escherichia coli* [216] have a higher yield of chlorogenic acid than the stem node culture of *M. nigra* elicited with CuSO_4_. To the best of our knowledge, there is currently no established system that would utilize microbes or fermentation to obtain 4-caffeoylquinic acid. Therefore, the yield of this compound obtained in the nodal culture of *M. nigra* cannot be compared with yields in other biotechnological processes. In the future, in addition to optimizing the method for the production of caffeoylquinic acids using the stem node culture of *M. nigra*, it would also be necessary to evaluate the economic advantages and disadvantages of this method.

## 4. Materials and Methods

### 4.1. Chemicals

Indole-3-butyric acid (IBA), metatopolin, Murashige and Skoog (MS) medium, myo-Inositol, plant agar, and sucrose were obtained from Duchefa (Haarlem, The Netherlands). Aluminum chloride hexahydrate, CuSO_4_, HPLC-grade acetonitrile, HPLC-grade methanol, sodium acetate (CH_3_COONa), and sodium carbonate (Na_2_CO_3_) were purchased from Honeywell (Charlotte, United States of America). Folin–Ciocalteu’s phenol reagent was supplied by Merck (Darmstadt, Germany). HPLC-grade formic acid, Trolox (6-hydroxy-2,5,7,8-tetramethylchroman-2-carboxylic acid), and standard compounds gallic acid, chlorogenic acid, kaempferol-3-O-glucoside, *p*-coumaric acid, quercetin-3-O-glucoside, and quercetin-3-O-rutinoside (rutin) were obtained from Sigma-Aldrich (Hamburg, Germany). A 2,2-diphenyl-1-picrylhydrazyl (DPPH) reagent was purchased from TCI (Tokyo, Japan). The ultra-pure water (resistance above 18 MΩ cm) used was obtained from a Milli-Q water purification system.

### 4.2. Plant Material

*M. nigra* plant material used in this experiment was obtained from the stem node culture collection of the Department of Botany and Plant Physiology, Faculty of Agriculture and Life Sciences, at the University of Maribor. It was originally established from the buds of the local old *M. nigra* tree from Osp, Slovenia (Lat.: 45.5721989° N; Long.: 13.8589273° E). It was grown and propagated on the MS medium [217]. The MS medium was enriched with 3% sucrose, 0.6% plant agar, 1 mg/L of IBA and 0.5 mg/L of metatopolin. Before autoclaving, the pH of the MS medium was adjusted to 5.7. Autoclaving was carried out at a temperature of 121 °C at a pressure of 1.2 bar for 20 min. Such an MS medium recipe is used at the Department of Botany and Plant Physiology, Faculty of Agriculture and Life Sciences, at the University of Maribor for micropropagation and subsequent rooting of mulberry stem node cultures, which allows the micropropagated material to be available for germplasm collections ex situ. Two shoots of approximately the same size and the same fresh weight (approx. 0.25 g) were placed under aseptic conditions on the surface of 25 mL of enriched MS medium in a 100 mL jar, which was immediately closed with a suitable lid. The experiment was repeated on 54 jars containing a total of 108 shoots. Six jars were sampled on the same day of the performed micropropagation in order to obtain information about the state of the observed parameters at day 0 of the experiment. The remaining glasses were divided into 4 equal-sized groups (12 glasses each). To the first (control) group, 2 mL of distilled water was aseptically added through a sterile filter, and to the treated groups, 2 mL of 0.5 mM, 1 mM, and 3 mM CuSO_4_ aqueous solution. The concentrations of the CuSO_4_ aqueous solution were selected based on a study by Park et al., which examined the elicitation of the adventitious root cultures of *Althaea officinalis* L. with Cu [25]. After 21 days, 6 glasses from each of the four groups were sampled. The remaining jars were observed for a further 21 days (42 days in total) and sampled at that time. The plant development, fresh weight, dry weight, individual phenolic compounds content, TPC, TFC, and antioxidant activity of the extracts were determined for the plantlets samples. Throughout the experiment, stem node cultures were grown under controlled conditions of 23 ± 2 °C, a photoperiod of 16 h at 38–50 μmol m^−2^ s^−1^, a spectrum range of 420–700 nm (Osram L 58W/77—Fluora, Munich, Germany), and 50% relative humidity.

Plant material was sampled following the instructions of Tausz et al. [218], with slight modifications. Immediately after removal from the MS medium, the plantlets were washed in distilled water to remove the MS medium and to remove the underground parts. In the shortest possible time, the samples were frozen in liquid nitrogen, later transferred to a freezer at −80 °C and subsequently freeze-dried (Lyophilizer Alpha, Christ, Osterode am Harz, Germany) and grounded (TissueLyser II, Qiagen, Hilden, Germany). The experiment, with 6 replicates per treatment, was repeated twice. The results of the two repetitions did not differ significantly from each other; therefore, the results of both replicates are presented together.

### 4.3. Plantlets Development

Plantlets growth and development were evaluated at day 0 of the experiment and then again at the 21st and 42nd day of the experiment. The assessment of the visual quality of the plantlets was based on observable characteristics, including variations in plantlet size, leaf color, and the appearance of chlorosis, necrosis, and deformations.

### 4.4. Fresh and Dry Biomass

The fresh and dry biomass content was determined at day 0 of the experiment, followed by measurements after 21 days and 42 days of the experiment across all four treatments.

### 4.5. Extraction Procedures

For the extraction, 1.5 mL of 70% methanol (acidified in 3% formic acid) was added to 20 mg of each lyophilized sample. The extraction procedure began by placing the mixture in an ultrasonic bath (Sonis 3, Iskra Pio). The duration of the sonication was set to 30 min and took place at 4 °C. After completion of the sonication, samples were centrifuged for 15 min (samples for TPC analysis) or 60 min (samples for HPLC analysis) at a speed of 12,000 rpm at 4 °C. After that, samples for HPLC analysis were filtered through 0.45 μm PTFE filters and transferred into vials, prior to injection into HPLC system. The extraction was carried out in duplicate. Both parallels were always analyzed in the subsequent spectrophotometric and HPLC analyses. The extracts were stored in a dark place at −20 °C until further analysis.

### 4.6. Determination of Total Phenolic Content in Plant Material

The Folin–Ciocalteu method, a common spectrophotometric method [219], was used to determine TPC in the acidified methanolic extracts. For the analysis, 100 μL of acidified methanolic extract was mixed with 200 μL of the Folin–Ciocalteu solution. After 3 min, 800 μL of the Na_2_CO_3_ solution (92.5 g L^−1^) was added. The mixture was vortexed and incubated for 2 h in the dark at room temperature on the orbital shaker (GyroTwister, Labnet, Edison, NJ, USA). Gallic acid-containing standard solutions were prepared in 70% methanol (acidified in 3% formic acid) at concentrations ranging from 0.052 to 0.280 mg mL^−1^ to generate the calibration curve. The absorbance of the standards and the samples were measured with a UV-ViS spectrophotometer (Varian Cary 50 Bio, Varian, Palo Alto, CA, USA) at 765 nm against a blank. TPC was expressed as milligrams of gallic acid equivalents per gram dry weight of plant material (mg GA g^−1^ DW). All samples were analyzed in duplicate.

### 4.7. Determination of Total Flavonoid Content in Plant Material

TFC was measured by a standard spectrophotometric method [220] with slight modifications [221]. For these analyses, 125 μL of acidified methanolic extracts was mixed with 375 μL of 70% methanol acidified with 3% formic acid. Afterwards 25 μL of AlCl_3_ (10%), 25 μL of CH_3_COONa (1.0 mol L^−1^), and 700 μL of ultra-pure water were added. The mixture was vortexed and incubated for 30 min in the dark at room temperature on the orbital shaker (GyroTwister, Labnet, Edison, NJ, USA). The absorbances of samples and standards were measured with a UV-ViS spectrophotometer (Varian Cary 50 Bio, Varian, Palo Alto, CA, USA) against a blank at 415 nm. Standard solutions of rutin in the concentration range from 10 to 100 mg L^−1^ were prepared in 70% methanol acidified with 3% formic acid. TFC was expressed as milligrams of rutin equivalents per gram dry weight of plant material (mg RUT g^−1^ DW). All samples were analyzed in duplicate.

### 4.8. HPLC Analysis of Phenolic Compounds in Plant Material

The HPLC analyses were performed using an HPLC-PDA chromatography system (HPLC Waters Alliance 2695 System, coupled with a 2996 photodiode array detector). The separation was performed on a column Gemini C18, Phenomenex (150 mm × 4.6 mm; 3 μm). The mobile phase A consisted of 0.1% formic acid/3% acetonitrile/96.9% ultra-pure water, and the mobile phase B consisted of 0.1% formic acid/3% ultra-pure water/96.9% acetonitrile, which were mixed according to the gradient method described by Mikulic-Petkovsek et al. [222]. Samples were eluted from 5% to 20% B in the first 15 min, followed by a linear gradient from 20% to 30% B for 5 min, then an isocratic mixture for 5 min, followed by a linear gradient from 30% to 90% B for 5 min, and then an isocratic mixture for 15 min, before returning to the initial conditions. The volume of injected samples was 10 μL and the flow rate of the mobile phase was set at 0.6 mL/min at a column temperature of 25 °C. Phenolic acids and flavonoids were detected at wavelengths of 280 nm and 350 nm.

The acidified methanolic extracts were also subjected to a mass spectrometer (LTQ XL Linear Ion Trap Mass Spectrometer, Thermo Fisher Scientific, Waltham, MA, USA) with an electrospray interface (ESI) operating in negative ion mode. The procedures were previously described by Mikulic-Petkovsek et al. [223]. The analyses were carried out using full-scan data-dependent MS^2^ scanning from *m*/*z* 110 to 1500. Column and chromatographic conditions were identical to those used for the HPLC–PDA analysis. The capillary temperature was 250 °C, the sheath gas and auxiliary gas were 60 and 15 units, respectively, the source voltage was 3 kV, and normalized collision energy was between 20 and 35%. Spectral data were elaborated using the XcaliburTM 4.3 software (Thermo Scientific, Waltham, MA, USA).

Each sample vial was analyzed twice. The identification of compounds was confirmed by comparing their spectra, retention times, and fragmentation, as well as by adding the standard solution to the sample. Quantification was achieved by comparing with the corresponding external standards (chlorogenic acid, kaempferol-3-O-glucoside *p*-coumaric acid, rutin, and quercetin-3-O-glucoside) of known concentration. The content of each determined compound was calculated from the peak area of the sample and with the corresponding standard curve. For the compounds for which the standards were not available, related compounds were used as standards. Therefore, 4-caffeoylquinic acid and *cis*-5-caffeoylquinic acid were quantified in the equivalent of chlorogenic acid (*trans*-5-caffeoylquinic acid) and *p*-coumaric acid hexoside 1, *p*-coumaric acid hexoside 2, *trans*-5-*p*-coumaroylquinic acid, *cis*-5-*p*-coumaroylquinic acid, and 3-*p*-coumaroylquinic acid in the equivalent of *p*-coumaric acid. Kaempferol dirhamnosyl-hexoside, kaempferol-rhamnosylhexoside, kaempferol acetyl-rhamnosyl-hexoside, and kaempferol acetyl-hexoside were quantified in kaempferol-3-O-glucoside equivalents and quercetin dirhamnosyl-hexoside, quercetin-rhamnosyl-hexoside, quercetin acetyl-rhamnosyl-hexoside, quercetin malonyl-hexoside, and quercetin acetyl-hexoside were quantified in quercetin-3-O-glucoside equivalents.

In this study, the method was validated for linearity, intra-day precision, inter-day precision, limit of detection (LOD), and LOQ. The linearity of standard curves was confirmed by a linear least squares regression (R^2^ > 0.9990) and the quality coefficient (QC < 5%). The intra-day and inter-day precisions were expressed as relative standard deviation. The validation parameters are presented in Table S7. The contents of individual compounds were expressed in mg/g DW. The yield of each individual phenolic compound was calculated based on the concentrations of it and dry weight that can be obtained in one liter of MS medium. The yield of determined phenolic compounds was expressed as the mg of compound/1 L of MS medium.

### 4.9. DPPH Radical Scavenging Activity of Antioxidants

The antioxidant activity of the extracts was determined using the standard DPPH spectrophotometric method with slight modifications according to Senekovič et al. [87]. For these analyses, 0.05 mL of acidified methanolic extracts were mixed with 4.95 mL of DPPH reagent. The mixture was vortexed and incubated for 30 min in the dark at room temperature on the orbital shaker (GyroTwister, Labnet, Edison, New Jersey, USA). The antioxidant activity of the standards and the samples was measured spectrophotometrically (Varian Cary 50 Bio spectrophotometer, Varian, Palo Alto, California, USA) at 517 nm against a blank. The antioxidant activity of the extracts and standards was calculated as millimoles Trolox equivalents per gram of dry weight of plant material (mmol Trolox g^−1^ DW), using the regression equation between the standard solutions of Trolox (0.2–5.0 mmol L^−1^ in 70% methanol acidified with 3% formic acid). All the samples were analyzed in duplicate.

### 4.10. Statistics

The plantlets biomass results, which were calculated from two duplicates, are represented by mean values (n = 24) and standard deviations (±SD) and were evaluated statistically by one-way analysis of variance (ANOVA) using the SPSS 21 software (SPSS Inc., Chicago, IL, USA). Significant differences between the mean values were determined using the Duncan post hoc test. Significant differences (*p* < 0.05) between the mean values were indicated by different letters. The results of the biochemical analyses (TPC, TFC, content of individual phenolic compounds, antioxidant activity, and yield of individual phenolic compounds) are given as mean values (±standard error, SE) of the analyses of plant material cultivated in two replicates. The measurements were performed at least twice for each sample and in duplicate. The Kolmogorov–Smirnov test was used to examine the normal distribution of data. One-way analysis of variance (ANOVA) was used to test for the differences between the effect of different cultivation conditions and biochemical traits (SPSS Inc., Chicago, IL, USA). The post hoc Duncan test was used for the biochemical traits that were evaluated as significant (*p* < 0.05). Two-way analysis of variance (ANOVA) was conducted to identify the cultivation time dependency and the effect of CuSO_4_ treatment on the biochemical traits of *M. nigra* stem node culture. (SPSS Inc., Chicago, IL, USA). The Pearson correlation coefficient was also calculated between evaluated chemical parameters (Past4).

## 5. Conclusions

The potential of *M. nigra* stem node culture for the production of phenolic compounds and the influence of elicitation with CuSO_4_ on this were investigated in this study. The results showed that the nodal cultures of *M. nigra* reached the highest fresh and dry weights when treated with 0.5 mM CuSO_4_ for 42 days. Moreover, these plantlets exhibited no visible symptoms of stress. During the 42 days of the experiment, the fresh and dry weights of plantlets grown under all treatments increased. The slowest growth was achieved by plantlets exposed to 3 mM CuSO_4_, which also had the highest degree of leaf chlorotic margins. The highest TPC and TFC were observed in plantlets treated with 3 mM CuSO_4_ for 42 days. This is the first study in which phenolic acids and flavonoids have been identified and quantified in *M. nigra* stem node culture. Among all quantified phenolic compounds, the main phenolic compound was chlorogenic acid. Among the *p*-coumaric acid derivatives, *p*-coumaric acid hexoside 1 was predominant, among the kaempferol derivatives, kaempferol acetyl-hexoside was predominant, while the main quercetin derivative was rutin. All predominant compounds reached their highest content in nodal cultures treated with 3 mM CuSO_4_ for 42 days as a response to copper stress, which coincides with the high DPPH antioxidant activity. Compared to some other plant species grown in vitro, *M. nigra* shows potential for the in vitro production of particular caffeoylquinic acids. For larger large-scale production, it would be necessary to investigate the elicitation with CuSO_4_ at more different concentrations and with more frequent sampling, which would give better insights into the temporal changes in the content of phenolics during stem node culture growth. It would also be necessary to test other elicitors that have been successful in other plant species and evaluate their impact on the biosynthesis of caffeoylquinic acids as well as on other valuable compounds in the nodal culture of *M. nigra*. Moreover, considering the strong biochemical response to CuSO_4_ exposure, it would be worthwhile to test the potential of *M. nigra* for use in phytoremediation in the future.

## Figures and Tables

**Table 1:** Mean content (±SE) of total phenolics (TPC, mg GA/g DW) and total flavonoids (TFC, mg RUT/g DW) in *M. nigra* grown in vitro (n = 24) in relation to different CuSO_4_ treatments, along with changes expressed as percentage increase/decrease compared to initial day 0. The different letters (a–i) indicate significant differences (*p* < 0.05), which were determined using a post hoc Duncan test.

Treatment	TPC (mg GA/g DW)			% Increase/Decrease	% Increase/Decrease	TFC (mg RUT/g DW)			% Increase/Decrease (Compared to Day 0)	% Increase/Decrease (Compared to Control Day)
0 days	7.069	±	0.030 ^g^	/	/	0.797	±	0.003 ^h^	/	/
Control, 21 days	6.943	±	0.116 ^g^	−1.78	/	0.916	±	0.003 ^g^	+14.93	/
0.5 mM CuSO_4,_ 21 days	8.952	±	0.008 ^e^	+26.64	+28.94	0.697	±	0.003 ^i^	−12.55	−23.91
1 mM CuSO_4,_ 21 days	9.168	±	0.007 ^e^	+29.69	+32.05	1.109	±	0.003 ^e^	+39.15	+21.07
3 mM CuSO_4,_ 21 days	11.451	±	0.004 ^b^	+61.99	+64.93	1.144	±	0.006 ^c^	+43.54	+24.89
Control, 42 days	7.879	±	0.008 ^f^	+11.46	/	0.994	±	0.004 ^f^	+24.72	/
0.5 mM CuSO_4,_ 42 days	10.113	±	0.004 ^d^	+43.06	+28.35	1.131	±	0.001 ^d^	+41.91	+13.78
1 mM CuSO_4,_ 42 days	10.385	±	0.008 ^c^	+46.91	+31.81	1.159	±	0.003 ^b^	+45.42	+16.60
3 mM CuSO_4,_ 42 days	14.249	±	0.214 ^a^	+101.57	+52.49	1.216	±	0.003 ^a^	+80.85	+22.33

**Table 2:** HPLC-PDA-MS^2^ data of the identified phenolic compounds in *M. nigra* stem node culture extracted by 70% MeOH acidified with 3% formic acid.

Retention Time [min]	Compound	[M − H]^−^ (*m*/*z*)	MS^2^ (*m*/*z*)	Identification Level [47] *
9.17	*p*-coumaric acid hexoside 1	325	163	2
11.91	*p*-coumaric acid hexoside 2	325	163	2
12.00	chlorogenic acid	353	191, 179	1
13.51	4-caffeoylquinic acid	353	173, 179, 191	2
14.10	*cis*-5-caffeoylquinic acid	353	191, 179	2
15.11	*trans*-5-*p*-coumaroylquinic acid	337	191, 163, 173	2
15.88	*cis*-5-*p*-coumaroylquinic acid	337	191, 163, 173	2
16.69	3-*p*-coumaroylquinic acid	337	163	2
18.09	quercetin dirhamnosyl-hexoside	755	609, 301	2
18.50	kaempferol dirhamnosyl-hexoside	739	593, 285	2
19.34	quercetin rhamnosyl-hexoside	609	301	2
20.51	rutin	609	301	1
21.01	quercetin-3-glucoside	463	301	1
21.55	quercetin acetyl-rhamnosyl-hexoside	651	463, 301	2
22.20	kaempferol rhamnosyl-hexoside	593	447, 285	2
23.07	kaempferol acetyl-rhamnosyl-hexoside	635	447, 285	2
23.27	quercetin malonyl-hexoside	549	463, 301	2
24.33	quercetin acetyl-hexoside	505	301	2
25.00	kaempferol acetyl-hexoside	489	285	2

* 1 = identified compound; 2 = putatively annotated compound; 3 = putatively characterized compound class; 4 = unknown compound.

**Table 3:** Mean contents (±SE) of caffeoylquinic acids (mg/g DW) in *M. nigra* stem node culture elicited with different concentrations of CuSO_4_ for different periods of time. Different letters (a–h) indicate significant differences (*p* < 0.05), which were determined using the post hoc Duncan test.

Treatment	Chlorogenic Acid			4-Caffeoylquinic Acid			*cis*-5-Caffeoylquinic Acid		
0 days	0.961	±	0.041 ^h^	0.672	±	0.029 ^g^	0.438	±	0.019 ^c^
Control, 21 days	1.225	±	0.020 ^g^	0.740	±	0.012 ^g^	0.398	±	0.007 ^de^
0.5 mM CuSO_4,_ 21 days	1.549	±	0.014 ^f^	0.856	±	0.008 ^f^	0.389	±	0.003 _e_
1 mM CuSO_4,_ 21 days	2.913	±	0.022 ^c^	1.605	±	0.012 ^d^	0.414	±	0.003 ^c–e^
3 mM CuSO_4,_ 21 days	4.364	±	0.152 ^b^	2.243	±	0.078 ^b^	0.510	±	0.018 ^b^
Control, 42 days	1.805	±	0.018 ^e^	0.941	±	0.010 ^f^	0.408	±	0.004 ^c–e^
0.5 mM CuSO_4,_ 42 days	2.272	±	0.090 ^d^	1.083	±	0.043 ^e^	0.419	±	0.017 ^c–e^
1 mM CuSO_4,_ 42 days	4.283	±	0.033 ^b^	2.038	±	0.016 ^c^	0.434	±	0.003 ^cd^
3 mM CuSO_4,_ 42 days	7.037	±	0.106 ^a^	3.122	±	0.047 ^a^	0.587	±	0.009 ^a^

**Table 4:** Mean contents (±SE) of *p*-coumaric acid derivatives (mg/g DW) in *M. nigra* stem node culture elicited with different concentrations of CuSO_4_ for different periods of time. Different letters (a–h) indicate significant differences (*p* < 0.05), which were determined using the post hoc Duncan test.

Treatment	*p*-Coumaric Acid Hexoside 1			*p*-Coumaric Acid Hexoside 2			*trans*-5-*p*-Coumaroylquinic Acid			*cis*-5-*p*-Coumaroylquinic Acid			3-*p*-Coumaroylquinic Acid		
0 days	0.120	±	0.005 ^h^	0.001	±	0.000 ^g^	0.001	±	0.000 ^f^	0.005	±	0.000 ^d^	<LOQ		
Control, 21 days	0.136	±	0.002 ^gh^	0.011	±	0.000 ^c^	0.005	±	0.000 ^e^	0.004	±	0.000 ^d^	0.001	±	0.000 ^b^
0.5 mM CuSO_4,_ 21 days	0.168	±	0.002 ^fg^	0.007	±	0.000 ^f^	0.006	±	0.000 ^e^	0.015	±	0.000 ^c^	<LOQ		
1 mM CuSO_4,_ 21 days	0.462	±	0.004 ^d^	0.009	±	0.000 ^de^	0.022	±	0.000 ^d^	0.026	±	0.000 ^b^	0.001	±	0.000 ^c^
3 mM CuSO_4,_ 21 days	0.747	±	0.026 ^b^	0.009	±	0.000 ^e^	0.038	±	0.001 ^c^	0.105	±	0.002 ^a^	0.001	±	0.000 ^b^
Control, 42 days	0.179	±	0.002 ^f^	0.015	±	0.000 ^a^	0.022	±	0.000 ^d^	0.003	±	0.000 ^d^	0.002	±	0.000 ^a^
0.5 mM CuSO_4,_ 42 days	0.219	±	0.009 ^e^	0.010	±	0.000 ^d^	0.025	±	0.001 ^d^	0.013	±	0.001 ^c^	0.001	±	0.000 ^b^
1 mM CuSO_4,_ 42 days	0.605	±	0.005 ^c^	0.013	±	0.000 ^b^	0.099	±	0.001 ^b^	0.023	±	0.000 ^b^	0.001	±	0.000 ^b^
3 mM CuSO_4,_ 42 days	1.071	±	0.016 ^a^	0.013	±	0.001 ^b^	0.192	±	0.003 ^a^	0.109	±	0.003 ^a^	0.001	±	0.000 ^b^

**Table 5:** Mean contents (±SE) of kaempferol derivatives (mg/g DW) in *M. nigra* stem node culture elicited with different concentrations of CuSO_4_ for different periods of time. Different letters (a–h) indicate significant differences (*p* < 0.05), which were determined using the post hoc Duncan test.

Treatment	Kaempferol Dirhamnosyl-Hexoside			Kaempferol Rhamnosyl-Hexoside			Kaempferol Acetyl-Rhamnosyl-Hexoside			Kaempferol Acetyl-Hexoside		
0 days	0.001	±	0.000 ^g^	0.011	±	0.000 ^a^	0.001	±	0.000 ^h^	0.003	±	0.000 ^f^
Control, 21 days	0.005	±	0.000 ^fg^	0.007	±	0.000 ^b^	0.003	±	0.000 ^g^	0.006	±	0.000 ^f^
0.5 mM CuSO_4,_ 21 days	0.009	±	0.000 ^f^	0.002	±	0.000 ^e^	0.003	±	0.000 ^g^	0.014	±	0.000 ^f^
1 mM CuSO_4,_ 21 days	0.026	±	0.000 ^e^	0.008	±	0.000 ^b^	0.010	±	0.000 ^f^	0.064	±	0.000 ^d^
3 mM CuSO_4,_ 21 days	0.057	±	0.002 ^c^	0.005	±	0.000 ^c^	0.013	±	0.001 ^e^	0.233	±	0.008 ^b^
Control, 42 days	0.027	±	0.000 ^e^	0.003	±	0.000 ^d^	0.020	±	0.000 ^d^	0.016	±	0.000 ^f^
0.5 mM CuSO_4,_ 42 days	0.047	±	0.002 ^d^	0.003	±	0.000 ^d^	0.023	±	0.001 ^c^	0.039	±	0.002 ^e^
1 mM CuSO_4,_ 42 days	0.134	±	0.001 ^b^	0.002	±	0.000 ^e^	0.044	±	0.000 ^b^	0.177	±	0.001 ^c^
3 mM CuSO_4,_ 42 days	0.319	±	0.005 ^a^	0.001	±	0.000 ^f^	0.071	±	0.005 ^a^	0.705	±	0.011 ^a^

**Table 6:** Mean contents (±SE) of quercetin derivatives (mg/g DW) in *M. nigra* stem node culture elicited with different concentrations of CuSO_4_ for different periods of time. Different letters (a–h) indicate significant differences (*p* < 0.05), which were determined using the post hoc Duncan test.

Treatment	Quercetin Dirhamnosyl-Hexoside			Quercetin Rhamnosyl-Hexoside			Rutin			Quercetin-3-Glucoside			Quercetin Acetyl-Rhamnosyl-Hexoside			Quercetin Malonyl-Hexoside			Quercetin Acetyl-Hexoside		
0 days	0.052	±	0.002 ^a^	0.001	±	0.000 ^f^	0.019	±	0.001 ^h^	0.007	±	0.000 ^g^	0.012	±	0.001 ^h^	0.055	±	0.002 ^f^	0.001	±	0.000 ^e^
Control, 21 days	0.041	±	0.001 ^b^	0.005	±	0.000 ^ef^	0.038	±	0.001 ^g^	0.010	±	0.000 ^f^	0.021	±	0.000 ^g^	0.057	±	0.001 ^f^	0.003	±	0.000 ^e^
0.5 mM CuSO_4,_ 21 days	0.029	±	0.000 ^d^	0.008	±	0.000 ^e^	0.048	±	0.000 ^f^	0.011	±	0.000 ^f^	0.027	±	0.000 ^f^	0.031	±	0.000 ^h^	0.003	±	0.000 ^e^
1 mM CuSO_4,_ 21 days	0.017	±	0.000 ^f^	0.022	±	0.000 ^d^	0.086	±	0.001 ^e^	0.025	±	0.000 ^d^	0.045	±	0.000 ^e^	0.092	±	0.001 ^d^	0.007	±	0.000 ^d^
3 mM CuSO_4,_ 21 days	0.011	±	0.000 ^g^	0.048	±	0.002 ^c^	0.129	±	0.005 ^c^	0.036	±	0.001 ^c^	0.067	±	0.002 ^c^	0.102	±	0.004 ^c^	0.009	±	0.000 ^d^
Control, 42 days	0.037	±	0.000 ^c^	0.024	±	0.000 ^d^	0.086	±	0.001 ^e^	0.016	±	0.000 ^e^	0.043	±	0.000 ^e^	0.067	±	0.001 ^e^	0.030	±	0.000 ^c^
0.5 mM CuSO_4,_ 42 days	0.026	±	0.001 ^e^	0.043	±	0.002 ^c^	0.110	±	0.004 ^d^	0.018	±	0.001 ^e^	0.054	±	0.002 ^d^	0.037	±	0.001 ^g^	0.032	±	0.001 ^c^
1 mM CuSO_4,_ 42 days	0.016	±	0.000 ^f^	0.115	±	0.001 ^b^	0.196	±	0.002 ^b^	0.042	±	0.000 ^b^	0.093	±	0.001 ^b^	0.109	±	0.001 ^b^	0.079	±	0.001 ^b^
3 mM CuSO_4,_ 42 days	0.011	±	0.000 ^g^	0.119	±	0.004 ^a^	0.322	±	0.005 ^a^	0.066	±	0.001 ^a^	0.150	±	0.002 ^a^	0.276	±	0.002 ^a^	0.117	±	0.002 ^a^

**Table 7:** Mean (±SE) DPPH inhibition (%) and mean (±SE) antioxidant activity of acidified methanolic extracts (mmol Trolox/g DW) of *M. nigra* grown in vitro (n = 24) in relation to different CuSO_4_ treatments and cultivation periods, together with the percentage increase/decrease. The different letters (a–f) indicate significant differences (*p* < 0.05), which were determined using the post hoc Duncan test.

Treatment	DPPH Inhibition (%)			DPPH Antioxidant Activity (mmol Trolox/g DW)			% Increase/Decrease (Compared to Day 0)	% Increase/Decrease (Compared to Control)
0 days	20.30	±	0.02 ^e^	0.0331	±	0.0001 ^e^	/	/
Control, 21 days	18.31	±	0.53 ^f^	0.0242	±	0.0024 ^f^	−26.89	/
0.5 mM CuSO_4,_ 21 days	21.30	±	0.01 ^d^	0.0376	±	0.0000 ^d^	+13.60	+55.37
1 mM CuSO_4,_ 21 days	24.39	±	0.07 ^c^	0.0515	±	0.0003 ^c^	+55.59	+112.81
3 mM CuSO_4,_ 21 days	26.59	±	0.11 ^b^	0.0614	±	0.0005 ^b^	+85.49	+153.72
Control, 42 days	20.67	±	0.04 ^e^	0.0348	±	0.0002 ^e^	+5.14	/
0.5 mM CuSO_4,_ 42 days	24.98	±	0.01 ^c^	0.0541	±	0.0000 ^c^	+63.44	+55.46
1 mM CuSO_4,_ 42 days	26.31	±	0.17 ^b^	0.0601	±	0.0008 ^b^	+81.57	+72.70
3 mM CuSO_4,_ 42 days	33.59	±	0.13 ^a^	0.0928	±	0.0006 ^a^	+180.36	+166.67
0.001 M chlorogenic acid	64.35			3.0801			/	/
0.001 M *p*-coumaric acid	49.32			2.1798			/	/
0.001 M kaempferol-3-glucoside	60.51			2.8501			/	/
0.001 M quercetin-3-glucoside	93.06			4.7998			/	/
0.001 M rutin	96.21			4.9884			/	/

**Table 8:** Pearson’s correlation coefficient between TPC, TFC, and content of groups of quantified individual phenolic compounds with respect to the DPPH antioxidant activity of acidified methanolic extracts. ** correlation is significant at the 0.01 level (2-tailed); * correlation is significant at the 0.05 level (2-tailed).

Observed Parameter	DPPH Antioxidant Activity
TPC	0.978 **
TFC	0.760 **
*p*-coumaric acid derivatives	0.941 **
caffeoylquinic acids	0.960 **
quercetin derivatives	0.918 **
kaempferol derivatives	0.918 **

## Data Availability

The data used to support the findings of this study can be made available by the corresponding author upon request.

## Supplementary Materials

The following supporting information can be downloaded at: https://www.mdpi.com/article/10.3390/plants14050766/s1, Supplementary Table S1. One-way ANOVA results on the effects of treatment on TPC, TFC of individual phenolic compounds, and antioxidant activity. Supplementary Table S2. Two-way ANOVA results on the effects of CuSO_4_ treatment and cultivation time on TPC, TFC, and content of individual phenolic compounds. Supplementary Table S3. The mean yields of caffeoylquinic acids. Supplementary Table S4. The mean yields of *p*-coumaric acid derivatives. Supplementary Table S5. The mean yields of kaempferol derivatives. Supplementary Table S6. The mean yields of quercetin derivatives. Supplementary Table S7. Method validation parameters.
