# Supplementary material for: Copper Sulfate Elicitation Effect on Biomass Production, Phenolic Compounds Accumulation, and Antioxidant Activity of Morus nigra L. Stem Node Culture
Source: Plants (Basel). 2025 Mar 2;14(5):766. doi: 10.3390/plants14050766 (PMC11901489; doi:10.3390/plants14050766)
Supplement: Supplementary file 1 [file plants-14-00766-s001.zip › plants-3478055-supplementary.pdf]

Table S1. One-way ANOVA test results for the effect of different cultivation conditions ($CuSO_4$ treatment and cultivation time) on the phenolic compounds composition in *M. nigra* stem node culture.

Observed parameter	Sum of squares	Df	F	Sig
TPC	85.898	8	801758	**<0.001**
TFC	0.518	8	2573297	**<0.001**
p-coumaric acid hexoside 1	1.798	8	931.513	**<0.001**
p-coumaric acid hexoside 2	1.800	8	416.535	**<0.001**
chlorogenic acid	62.291	8	750.728	**<0.001**
4-caffeoylquinic acid	11.289	8	543.505	**<0.001**
cis-5-caffeoylquinic acid	0.066	8	33.293	**<0.001**
trans-5-*p*-coumaroylquinic acid	0.062	8	2988.894	**<0.001**
cis-5-*p*-doumaroylquinic acid	0.029	8	929.897	**<0.001**
3-*p*-coumaroylquinic acid	0.000	8	632.575	**<0.001**
quercetin dirhamnosyl-hexoside	0.003	8	272.720	**<0.001**
kaempferol dirhamnosyl-hexoside	0.166	8	2962.373	**<0.001**
quercetin rhamnosyl-hexoside	0.124	8	2936.922	**<0.001**
rutin	0.142	8	1184.338	**<0.001**
quercetin-3-glucoside	0.006	8	993.197	**<0.001**
quercetin acetyl-rhamnosyl-hexoside	0.029	8	1003.825	**<0.001**
kaempferol rhamnosyl-hexoside	0.000	8	370.002	**<0.001**
kaempferol acetyl-rhamnosyl-hexoside	0.009	8	2060.431	**<0.001**
quercetin malonyl-hexoside	0.016	8	349.013	**<0.001**
quercetin acetyl-hexoside	0.027	8	2852.790	**<0.001**
kaempferol acetyl-hexoside	0.826	8	2540.431	**<0.001**
antioxidant activity	0.007	8	545.186	**<0.001**

Table S2. Two-way ANOVA test results for the effect of treatment with $CuSO_4$ and cultivation time on the phenolic compounds synthesis in *M. nigra* stem node culture.

	Sum of squares	Df	F	Sig
**TPC**				
$CuSO_4$	60.196	3	1.498.294	**<0.001**
cultivation time	9.496	2	354.532	**<0.001**
$CuSO_4$ * cultivation time	2.192	3	54.562	**<0.001**
**TFC**				
$CuSO_4$	0.206	3	2.723.890	**<0.001**
cultivation time	0.134	2	2.657.829	**<0.001**
$CuSO_4$ * cultivation time	0.102	3	1.348.545	**<0.001**
***p*-coumaric acid hexoside 1**				
$CuSO_4$	1.476	3	2.039.802	**<0.001**
cultivation time	0.080	2	166.312	**<0.001**
CuSO4 * cultivation time	0.051	3	70.928	**<0.001**
***p*-coumaric acid hexoside 2**				
$CuSO_4$	4,56E-05	3	182.325	**<0.001**

cultivation time	0	2	1.536.575	**<0.001**
$CuSO_4$ * cultivation time	1,04E-06	3	4.169	**0.042**
**chlorogenic acid**				
$CuSO_4$	43.648	3	1.402.806	**<0.001**
cultivation time	7.553	2	364.130	**<0.001**
$CuSO_4$ * cultivation time	2.737	3	87.958	**<0.001**
**4-caffeoylquinic acid**				
$CuSO_4$	8.777	3	1.126.773	**<0.001**
cultivation time	0.795	2	153.067	**<0.001**
$CuSO_4$ * cultivation time	0.295	3	37.869	**<0.001**
***cis*-5-caffeoylquinic acid**				
CuSO4	0.058	3	78.780	**<0.001**
cultivation time	0.006	2	12.723	**0.002**
CuSO4 * cultivation time	0.003	3	3.583	0.060
***trans*-5-*p*-coumaroylquinic acid**				
$CuSO_4$	0.027	3	3.515.059	**<0.001**
cultivation time	0.018	2	3.489.670	**<0.001**
$CuSO_4$ * cultivation time	0.012	3	1.586.415	**<0.001**
***cis*-5-*p*-coumaroylquinic acid**				
$CuSO_4$	0.027	3	2.317.983	**<0.001**
cultivation time	2,59E-05	2	3.351	0.082
$CuSO_4$ * cultivation time	7,11E-06	3	0.613	0.623
**3-*p*-coumaroylquinic acid**				
$CuSO_4$	2,14E-06	3	737.788	**<0.001**
cultivation time	3,09E-06	2	1.595.953	**<0.001**
$CuSO_4$ * cultivation time	9,12E-07	3	313.618	**<0.001**
**quercetin dirhamnosyl-hexoside**				
$CuSO_4$	0.002	3	403.567	**<0.001**
cultivation time	0.000	2	80.442	**<0.001**
$CuSO_4$ * cultivation time	8,25E-06	3	1.817	0.214
**kaempferol dirhamnosyl-hexoside**				
$CuSO_4$	0.074	3	3.495.512	**<0.001**
cultivation time	0.046	2	3.305.353	**<0.001**
$CuSO_4$ * cultivation time	0.036	3	1.713.081	**<0.001**
**quercetin rhamnosyl-hexoside**				
$CuSO_4$	0.054	3	3.406.169	**<0.001**
Cultivation time	0.035	2	3.359.902	**<0.001**
$CuSO_4$ * Cultivation time	0.027	3	1.702.777	**<0.001**
**rutin**				
$CuSO_4$	0.066	3	1.460.507	**<0.001**
cultivation time	0.045	2	1.508.146	**<0.001**
$CuSO_4$ * cultivation time	0.013	3	285.970	**<0.001**
**quercetin-3-glucoside**				
$CuSO_4$	0.004	3	1.729.300	**<0.001**
cultivation time	0.001	2	640.985	**<0.001**
$CuSO_4$ * cultivation time	0.000	3	163.399	**<0.001**
**quercetin acetyl-rhamnosyl-hexoside**				

$CuSO_4$	0.014	3	1.309.449	**<0.001**
cultivation time	0.009	2	1.188.072	**<0.001**
$CuSO_4$ * cultivation time	0.002	3	209.037	**<0.001**
**kaempferol rhamnosyl-hexoside**				
$CuSO_4$	2,25E-05	3	119.443	**<0.001**
cultivation time	8,96E-05	2	713.057	**<0.001**
$CuSO_4$ * cultivation time	2,96E-05	3	156.930	**<0.001**
**kaempferol acetyl-rhamnosyl-hexoside**				
$CuSO_4$	0.002	3	1.568.275	**<0.001**
cultivation time	0.004	2	4.175.228	**<0.001**
$CuSO_4$ * cultivation time	0.001	3	669.183	**<0.001**
**quercetin malonyl-hexoside**				
$CuSO_4$	0.015	3	845.535	**<0.001**
cultivation time	0.001	2	55.239	**<0.001**
$CuSO_4$ * cultivation time	7,81E-05	3	4.503	**0.034**
**quercetin acetyl-hexoside**				
$CuSO_4$	0.006	3	1.736.944	**<0.001**
cultivation time	0.015	2	6.128.510	**<0.001**
$CuSO_4$ * cultivation time	0.005	3	1.293.179	**<0.001**
**kaempferol acetyl-hexoside**				
$CuSO_4$	0.548	3	4.492.037	**<0.001**
cultivation time	0.096	2	1.183.555	**<0.001**
$CuSO_4$ * cultivation time	0.140	3	1.148.992	**<0.001**

Table S3. Mean yields (±SE) of quantified caffeoylquinic acids (mg/L MS medium) obtained from *M. nigra* grown *in vitro* (n = 24) per 1 L of prepared MS medium in relation to different $CuSO_4$ treatments. The different letters (a–i) indicate significant differences ($p < 0.05$), which were determined using the post hoc Duncan test.

Treatment	chlorogenic acid	4-caffeoylquinic acid	*cis*-5-caffeoylquinic acid
0 day	0.8341 ± 0.0354^{i}	0.5833 ± 0.0248^{h}	0.3802 ± 0.0161^{f}
Control 21 days	3.2238 ± 0.0539^{h}	1.9469 ± 0.0325^{g}	1.0487 ± 0.0175^{d}
0.5 mM $CuSO_4$ 21 days	4.7344 ± 0.0423^{g}	2.6144 ± 0.0234^{f}	1.1898 ± 0.0106^{c}
1 mM $CuSO_4$ 21 days	6.4077 ± 0.0489^{f}	3.5314 ± 0.0270^{e}	0.9100 ± 0.0069^{e}
3 mM $CuSO_4$ 21 days	8.6931 ± 0.3036^{d}	4.4681 ± 0.1561^{c}	1.0159 ± 0.0355^{d}
Control 42 days	7.4274 ± 0.0754^{e}	3.8718 ± 0.0393^{d}	1.6793 ± 0.0171^{b}
0.5 mM $CuSO_4$ 42 days	9.5242 ± 0.3767^{c}	4.5399 ± 0.1796^{c}	1.7564 ± 0.0695^{ab}
1 mM $CuSO_4$ 42 days	16.3611 ± 0.1260^{b}	7.7852 ± 0.0600^{b}	1.6579 ± 0.0128^{b}
3 mM $CuSO_4$ 42 days	21.3362 ± 0.3204^{a}	9.4659 ± 0.1422^{a}	1.7798 ± 0.0267^{a}

Table S4. Mean yields (±SE) of quantified *p*-coumaric acid derivatives (mg/L MS medium) obtained from *M. nigra* grown *in vitro* (n = 24) per 1 L of prepared MS medium in relation to different $CuSO_4$ treatments. The different letters (a–i) indicate significant differences ($p < 0.05$), which were determined using the post hoc Duncan test.

Treatment	*p*-coumaric acid hexoside 1	*p*-coumaric acid hexoside 2	*trans*-5-*p*-coumaroylquinic acid	*cis*-5-*p*-coumaroylquinic acid	3-*p*-coumaroylquinic acid
0 day	0.1042 ± 0.0044^{i}	0.0009 ± 0.0000^{h}	0.0009 ± 0.0000^{h}	0.0043 ± 0.0002^{f}	<LOQ
Control 21 days	0.3590 ± 0.0060^{h}	0.0300 ± 0.0005^{e}	0.0126 ± 0.0002^{g}	0.0093 ± 0.0002^{f}	0.0026 ± 0.0000^{e}
0.5 mM $CuSO_4$ 21 days	0.5134 ± 0.0065^{g}	0.0222 ± 0.0002^{f}	0.0169 ± 0.0002^{g}	0.0459 ± 0.0004^{e}	<LOQ
1 mM $CuSO_4$ 21 days	1.0175 ± 0.0078^{d}	0.0207 ± 0.0002^{f}	0.0477 ± 0.0004^{f}	0.0572 ± 0.0004^{d}	0.0013 ± 0.0000^{g}
3 mM $CuSO_4$ 21 days	1.4880 ± 0.0520^{c}	0.0179 ± 0.0006^{g}	0.0757 ± 0.0026^{e}	0.2171 ± 0.0076^{b}	0.0020 ± 0.0001^{f}
Control 42 days	0.7355 ± 0.0075^{f}	0.0634 ± 0.0006^{a}	0.0911 ± 0.0009^{d}	0.0130 ± 0.0001^{f}	0.0095 ± 0.0001^{a}
0.5 mM $CuSO_4$ 42 days	0.9180 ± 0.0363^{e}	0.0419 ± 0.0017^{c}	0.1048 ± 0.0041^{c}	0.0545 ± 0.0022^{de}	0.0042 ± 0.0002^{b}
1 mM $CuSO_4$ 42 days	2.3111 ± 0.0178^{b}	0.0497 ± 0.0004^{b}	0.3782 ± 0.0029^{b}	0.0879 ± 0.0007^{c}	0.0038 ± 0.0000^{c}
3 mM $CuSO_4$ 42 days	3.2473 ± 0.0488^{a}	0.0394 ± 0.0008^{d}	0.5821 ± 0.0087^{a}	0.3184 ± 0.0048^{a}	0.0030 ± 0.0000^{d}

Table S5. Mean yields (±SE) of quantified kaempferol derivatives (mg/L MS medium) obtained from *M. nigra* grown *in vitro* (n = 24) per 1 L of prepared MS medium in relation to different $CuSO_4$ treatments. The different letters (a–i) indicate significant differences ($p < 0.05$), which were determined using the post hoc Duncan test.

**Treatment**	**kaempferol dirhamnosyl-hexoside**	**kaempferol rhamnosyl-hexoside**	**kaempferol acetyl-rhamnosyl-hexoside**	**kaempferol acetyl-hexoside**
0 day	0.0009 ± 0.0000^{g}	0.0095 ± 0.0004^{e}	0.0009 ± 0.0000^{g}	0.0026 ± 0.0001^{f}
Control 21 days	0.0142 ± 0.0002^{fg}	0.0192 ± 0.0003^{a}	0.0080 ± 0.0001^{f}	0.0157 ± 0.0003^{f}
0.5 mM $CuSO_4$ 21 days	0.0283 ± 0.0003^{f}	0.0052 ± 0.0000^{g}	0.0082 ± 0.0000^{f}	0.0432 ± 0.0004^{ef}
1 mM $CuSO_4$ 21 days	0.0583 ± 0.0004^{e}	0.0173 ± 0.0001^{b}	0.0216 ± 0.0002^{e}	0.1414 ± 0.0011^{d}
3 mM $CuSO_4$ 21 days	0.1135 ± 0.0040^{d}	0.0100 ± 0.0005^{de}	0.0259 ± 0.0009^{e}	0.4641 ± 0.0162^{c}
Control 42 days	0.1124 ± 0.0011^{d}	0.0116 ± 0.0001^{d}	0.0823 ± 0.0008^{d}	0.0678 ± 0.0007^{e}
0.5 mM $CuSO_4$ 42 days	0.1970 ± 0.0078^{c}	0.0126 ± 0.0005^{c}	0.0964 ± 0.0038^{c}	0.1635 ± 0.0065^{d}
1 mM $CuSO_4$ 42 days	0.5119 ± 0.0039^{b}	0.0076 ± 0.0001^{f}	0.1681 ± 0.0013^{b}	0.6761 ± 0.0052^{b}
3 mM $CuSO_4$ 42 days	0.9672 ± 0.0145^{a}	0.0030 ± 0.0000^{h}	0.2153 ± 0.0032^{a}	2.1376 ± 0.0321^{a}

Table S6. Mean yields (±SE) of quantified quercetin derivatives (mg/L MS medium) obtained from *M. nigra* grown *in vitro* (n = 24) per 1 L of prepared MS medium in relation to different $CuSO_4$ treatments. The different letters (a–i) indicate significant differences ($p < 0.05$), which were determined using the post hoc Duncan test.

**Treatment**	**quercetin dirhamnosyl-hexoside**	**quercetin rhamnosyl-hexoside**	**rutin**	**quercetin-3-glucoside**	**quercetin acetyl-rhamnosyl-hexoside**	**quercetin malonyl-hexoside**	**quercetin acetyl-hexoside**
0 day	0.0451 ± 0.0019^{e}	0.0009 ± 0.0000^{h}	0.0165 ± 0.0007^{i}	0.0061 ± 0.0003^{h}	0.0104 ± 0.0004^{i}	0.0477 ± 0.0020^{g}	0.0009 ± 0.0001^{g}
Control 21 days	0.1071 ± 0.0018^{b}	0.0121 ± 0.0002^{g}	0.0997 ± 0.0017^{h}	0.0254 ± 0.0004^{g}	0.0555 ± 0.0009^{h}	0.1494 ± 0.0025^{e}	0.0070 ± 0.0001^{fg}
0.5 mM $CuSO_4$ 21 days	0.0885 ± 0.0008^{c}	0.0253 ± 0.0002^{f}	0.1480 ± 0.0013^{g}	0.0337 ± 0.0003^{f}	0.0813 ± 0.0007^{g}	0.0960 ± 0.0009^{f}	0.0085 ± 0.0001^{fg}
1 mM $CuSO_4$ 21 days	0.0376 ± 0.0003^{f}	0.0482 ± 0.0004^{e}	0.1892 ± 0.0014^{f}	0.0551 ± 0.0004^{e}	0.1000 ± 0.0008^{f}	0.2024 ± 0.0015^{d}	0.0152 ± 0.0001^{ef}
3 mM $CuSO_4$ 21 days	0.0219 ± 0.0008^{g}	0.0956 ± 0.0033^{d}	0.2570 ± 0.0090^{e}	0.0717 ± 0.0025^{cd}	0.1335 ± 0.0047^{e}	0.2032 ± 0.0071^{d}	0.0179 ± 0.0006^{e}
Control 42 days	0.1528 ± 0.0016^{a}	0.1002 ± 0.0010^{d}	0.3555 ± 0.0036^{d}	0.0664 ± 0.0007^{d}	0.1779 ± 0.0018^{d}	0.2762 ± 0.0028^{c}	0.1255 ± 0.0013^{d}
0.5 mM $CuSO_4$ 42 days	0.1090 ± 0.0043^{b}	0.1803 ± 0.0071^{c}	0.4611 ± 0.0182^{c}	0.0755 ± 0.0030^{c}	0.2264 ± 0.0090^{c}	0.1551 ± 0.0061^{e}	0.1341 ± 0.0053^{c}
1 mM $CuSO_4$ 42 days	0.0611 ± 0.0005^{d}	0.4393 ± 0.0048^{a}	0.7487 ± 0.0058^{b}	0.1604 ± 0.0012^{b}	0.3553 ± 0.0039^{b}	0.4164 ± 0.0032^{b}	0.3018 ± 0.0033^{b}
3 mM $CuSO_4$ 42 days	0.0334 ± 0.0005^{f}	0.3608 ± 0.0077^{b}	0.9763 ± 0.0147^{a}	0.2001 ± 0.0043^{a}	0.4548 ± 0.0068^{a}	0.8368 ± 0.0178^{a}	0.3547 ± 0.0075^{a}

Table S7. Validation parameters for HPLC-PDA method.

Standard compound	Concentration range [mg/L]	Regression equation	R^2	QC [%]	LOD [mg/L]	LOQ [mg/L]	Intra-Day precision [%RSD]	Inter-Day precision [%RSD]
chlorogenic acid	0.0624 - 0.208	$y = 98112866.8x - 404337.3$	0.9944	4.43	0.0159	0.0481	1.84	2.54
p-coumaric acid	0.00132 - 0.066	$y= 295270811.5x - 708397.9$	0.9984	4.28	0.0034	0.0102	0.73	1.90
kaempferol-3-O-glucoside	0.0042 - 0.042	$y = 110152194.8x + 286509.4$	0.9997	1.23	0.0011	0.0035	0.52	1.65
quercetin-3-O-glucoside	0.0075 - 0.0200	$y = 83653006.9x + 19257.3$	0.9939	4.72	0.0020	0.0060	0.56	1.33
rutin	0.0144 - 0.0576	$y = 28351216.7x - 44733.5$	0.9989	1.54	0.0019	0.0057	1.22	2.13
